# VirSorter2: a multi-classifier, expert-guided approach to detect diverse DNA and RNA viruses

**DOI:** 10.1186/s40168-020-00990-y

**Published:** 2021-02-01

**Authors:** Jiarong Guo, Ben Bolduc, Ahmed A. Zayed, Arvind Varsani, Guillermo Dominguez-Huerta, Tom O. Delmont, Akbar Adjie Pratama, M. Consuelo Gazitúa, Dean Vik, Matthew B. Sullivan, Simon Roux

**Affiliations:** 1 Department of Microbiology, Ohio State University, Columbus, OH 43210 USA; 2 The Biodesign Center for Fundamental and Applied Microbiomics, Center for Evolution and Medicine, School of Life Sciences, Arizona State University, Tempe, AZ 85287 USA; 3 Structural Biology Research Unit, Department of Integrative Biomedical Sciences, University of Cape Town, Observatory, Cape Town, 7701 South Africa; 4 Génomique Métabolique, Genoscope, Institut François Jacob, CEA, CNRS, Univ Evry, Université Paris-Saclay, 91057 Evry, France; 5 Viromica, 7870582 Santiago, Chile; 6 Civil, Environmental and Geodetic Engineering, Ohio State University, Columbus, OH 43210 USA; 7 Center of Microbiome Science, Ohio State University, Columbus, OH 43210 USA; 8 DOE Joint Genome Institute, Lawrence Berkeley National Laboratory, Berkeley, CA 94720 USA

## Abstract

**Background:**

Viruses are a significant player in many biosphere and human ecosystems, but most signals remain “hidden” in metagenomic/metatranscriptomic sequence datasets due to the lack of universal gene markers, database representatives, and insufficiently advanced identification tools.

**Results:**

Here, we introduce VirSorter2, a DNA and RNA virus identification tool that leverages genome-informed database advances across a collection of customized automatic classifiers to improve the accuracy and range of virus sequence detection. When benchmarked against genomes from both isolated and uncultivated viruses, VirSorter2 uniquely performed consistently with high accuracy (F1-score > 0.8) across viral diversity, while all other tools under-detected viruses outside of the group most represented in reference databases (i.e., those in the order *Caudovirales*). Among the tools evaluated, VirSorter2 was also uniquely able to minimize errors associated with atypical cellular sequences including eukaryotic genomes and plasmids. Finally, as the virosphere exploration unravels novel viral sequences, VirSorter2’s modular design makes it inherently able to expand to new types of viruses via the design of new classifiers to maintain maximal sensitivity and specificity.

**Conclusion:**

With multi-classifier and modular design, VirSorter2 demonstrates higher overall accuracy across major viral groups and will advance our knowledge of virus evolution, diversity, and virus-microbe interaction in various ecosystems. Source code of VirSorter2 is freely available (https://bitbucket.org/MAVERICLab/virsorter2), and VirSorter2 is also available both on bioconda and as an iVirus app on CyVerse (https://de.cyverse.org/de).

Video abstract

**Supplementary Information:**

The online version contains supplementary material available at 10.1186/s40168-020-00990-y.

## Introduction

Microbes are now widely recognized as driving nutrient and energy cycles that fuel marine and terrestrial ecosystems [1, 2], directly influencing human health and disease, and controlling the output of engineered ecosystems [3]. This explosive paradigm shift of our perspective of microbes is derived in large part from an ability to identify the “unculturable majority.” As high-throughput gene marker and metagenomic sequencing technologies have advanced, the true taxonomic and functional diversity of microbial communities could progressively be better explored [4–6]. Discovery and identification of viral sequences was at the forefront of the metagenomic revolution [7–9], but early studies were plagued by the lack of marker genes and an inability to “count” appropriate units in viral sequence space [10–12]. Fortunately, relevant “units” of viral diversity, at least for dsDNA viruses, are now routinely accessible through the de novo assembly of viral genomes from metagenomes. Thus the estimated 10^31^ viruses on the planet [13, 14] are being rapidly surveyed across soil, ocean, and human microbiomes, commonly yielding thousands to hundreds of thousands of dsDNA viruses in a single study [12, 15–19]. These large-scale surveys have helped implicate viruses as key microbiome regulators infecting ecologically critical microbes, impacting biogeochemical cycles, and altering evolutionary trajectories through horizontal gene transfer [20, 21]. Beyond viruses of bacteria and archaea, vast stores of previously unidentified viruses that infect eukaryotes including ssDNA viruses [22], RNA viruses [23–25], and giant viruses (the phylum *Nucleocytoviricota*, also known as NucleoCytoplasmic Large DNA Viruses [NCLDV]) [19, 26] are being identified, but are still in need of analytical approaches for systematic identification.

Given the sheer magnitude and significant importance of the virosphere across diverse ecosystems, establishing a broad genomic catalog of Earth’s viral diversity is critical. These efforts will rely on automated detection of viral genomes across a broad range of sequencing datasets [9]. Currently, two general computational approaches exist to identifying viral sequences. One set of tools rely on a combination of gene content and genomic structural features to distinguish viral from microbial sequences, including Prophinder [27], PhiSpy [28], VirSorter [29], the Earth’s Virome pipeline [17], PHASTER [30], MARVEL [31], and VIBRANT [32]. These genomic features are either statistically compared to a null model (Prophinder, VirSorter, PHASTER), or more recently have been used as input for automatic machine-learning classifiers (MARVEL and VIBRANT). The other approach uses the frequencies of DNA “words” (i.e., k-mers) found in known viral and cellular genomes as signatures to train machine-learning classifiers to recognize new viral and microbial sequences (e.g., VirFinder and DeepVirFinder [33, 34]). Both approaches efficiently detect common viruses that are well represented in databases, such as dsDNA bacteriophages from the *Caudovirales* order [31, 32], but they struggle with less well-documented viruses like ssDNA viruses [35], RNA viruses [36, 37], and viruses that infect archaea [38, 39]. One reason for this is that current approaches consider viruses as a single cohesive group for detection purposes, which is potentially problematic given the varied ecological and evolutionary rules that govern the diversity and evolution of different viral genomes [40]. While some features may span the virosphere (e.g., enrichment of uncharacterized genes, relative to microbes), others are group-specific (e.g., hallmark genes, specific genomic structure, or the presence of metabolic genes). Though machine learning approaches based on nucleotide composition would not suffer as much from viral database representation, they tend to confuse any unusual sequence as viral (e.g., plasmids or eukaryotic genome fragments [39]). Together, these observations call for the field to move beyond a single model to represent the virosphere for virus identification.

Here, we develop and introduce a new viral sequence identification tool, VirSorter2, which leverages recent sequencing efforts for under-represented viral groups to develop customized automatic classifiers that improve detection of viruses in the order *Caudovirales* (the focus of the original VirSorter tool [29]), while also identifying other major virus groups across a broad range of hosts, genome lengths, and genome complexity. VirSorter2 is designed modularly, enabling easy update of reference databases and individual classifiers as new viral groups are progressively described and characterized.

## Results and discussion

### The VirSorter2 framework

**Fig. 1:**
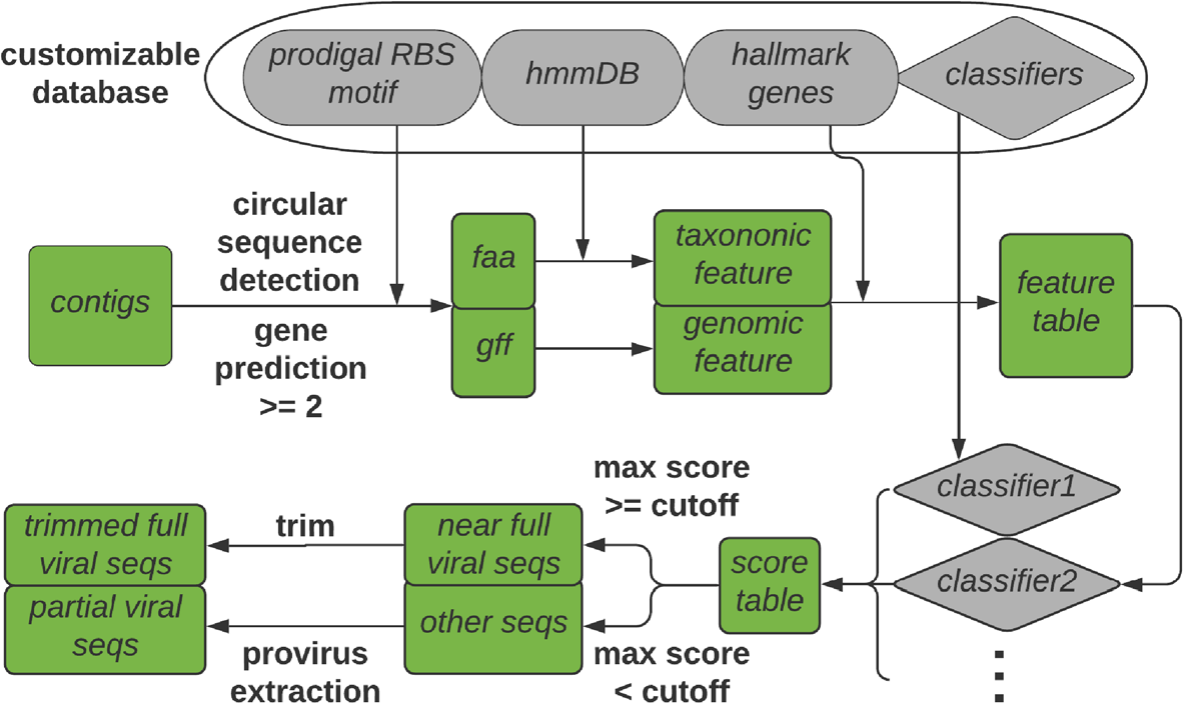
Overview of the VirSorter2 framework. Schematic of the viral prediction pipeline used in VirSorter2. “hmmDB” represents databases of HMM profiles including viral HMMs from Xfam (described in the “Methods” section) and viral protein families (VPF) from JGI Earth’s Virome [17], and cellular HMMs (archaeal, bacterial, eukaryotic) as well as “mixed” HMMs (not specific to either virus or cellular organisms) from Pfam [43]. A default cutoff of 30 is used for the HMM searches. “Classifiers” refers to random forest classifiers trained on known viral genomes and cellular genomes from different viral groups (see “Training classifiers” section in “Methods”). The default max score cutoff is set to 0.5

Viral sequence identification in VirSorter2 occurs via three steps: (i) input sequences are automatically annotated and relevant features are extracted, (ii) each sequence is scored independently using a set of classifiers customized for individual viral groups, and (iii) these scores are aggregated into a single prediction provided to the user (Fig. 1). Annotation of the input sequences follows current standards in the field [9] including coding sequence (CDS) identification with Prodigal (version 2.6.3) [41], and annotation of predicted CDS using HMMER3 (version 3.3) [42] against Pfam (release 32.0) [43] and a custom comprehensive viral HMM database including Xfams (described in the “Methods” section) and viral protein families (VPF) from the JGI Earth’s virome project [17]. Within this custom viral HMM database, profiles corresponding to viral hallmark genes were manually identified based on functional annotation and distribution across viral and microbial genomes for different viral groups. These include structural genes such as major capsid protein (MCP) or terminase large subunit for viruses in the order *Caudovirales* (as done in the original VirSorter); RNA-dependent RNA polymerase (RdRP) for the viral kingdom *Orthornavirae* (RNA viruses); MCP for viruses in the family *Microviridae*; replication-associated protein for viruses in the phylum *Cressdnaviricota* [44]; ATPase for viruses in the family *Inoviridae*; MCP, pATPase, primase, transcription elongation factor (TFIIS), and viral late gene transcription factor (VLTF3) for viruses in the phylum *Nucleocytoviricota* (i.e., nucleocytoplasmic large DNA viruses; NCLDVs); and MCP for viruses in the family *Lavidaviridae* (virophages) [40].

**Fig. 2:**
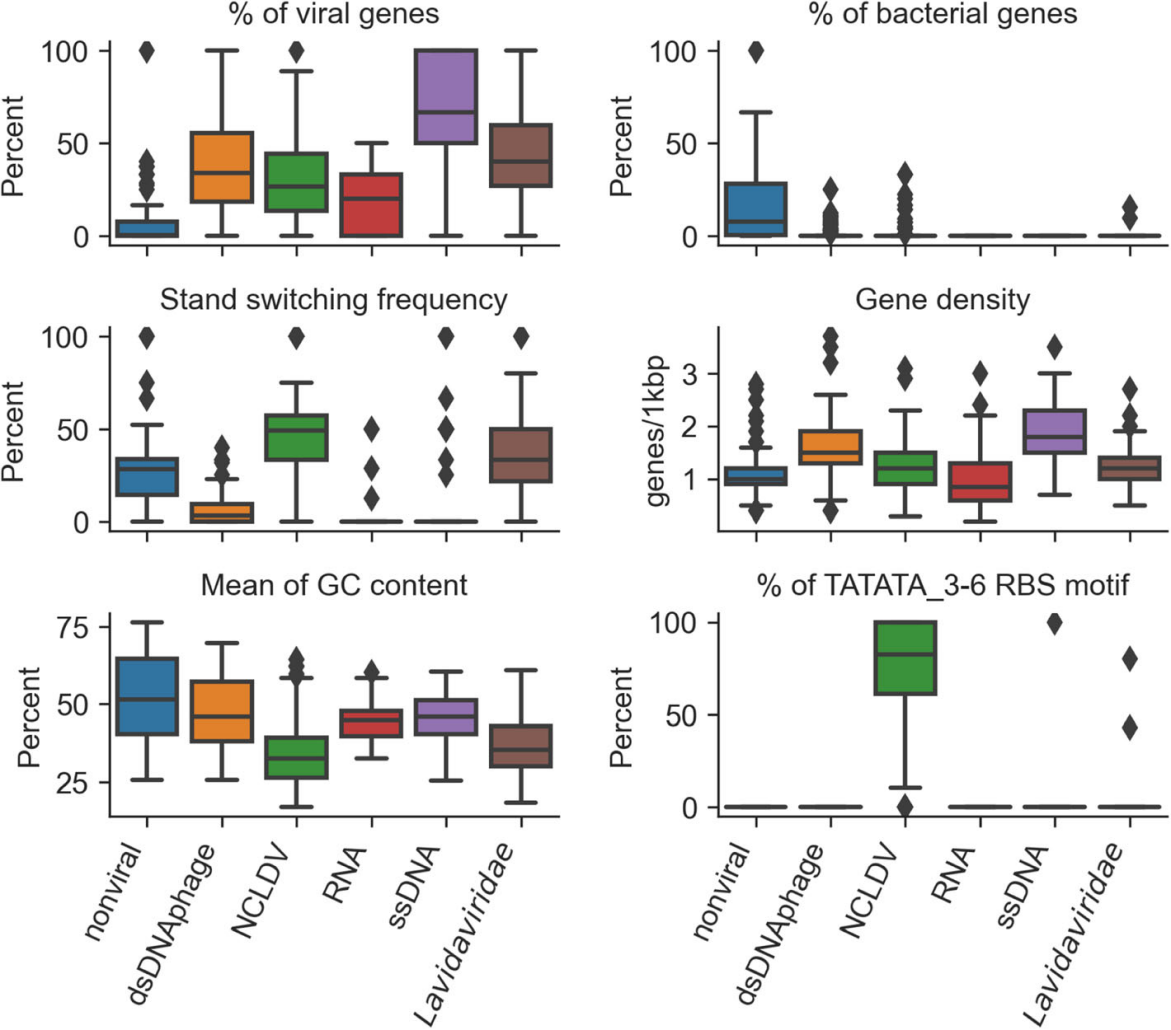
Boxplot of different features across non-viral and viral groups. “Nonviral” includes bacteria and archaea, fungi and protozoa, and plasmids. A subset of 100 random genome fragments were used for each group. “% of viral gene” is calculated as the percent of genes annotated as viral (best hit to viral HMMs) of all genes; “% of bacterial gene” is calculated as the percent of genes annotated as bacterial (best hit to bacterial HMMs) of all genes; “Strand switch frequency” is the percent of genes located on a different strand from the upstream gene (scanning from 5′ to 3′ in the + strand); “Gene density” is the average number of genes in every 1000 bp sequence (total number of genes divided by contig length and then multiplied by 1000); “Average GC content of genes” is the mean of GC content of all genes in a contig; “TATATA_3-6 motif frequency” is the percent of ribosomal binding sites (RBS) with “TATATA_3-6” motif

Different features were extracted from these annotations (Table 1 and Fig. 2). These features were then used as input for five distinct random forest classifiers, each associated with a different major type of viral group. These classifiers were trained on reference virus sequences from NCBI RefSeq and high quality genomes from new unpublished isolates (ssDNA) [22], metagenomes [15, 16, 19, 26, 45–47], and proviruses, i.e., viral genomes residing in bacteria or archaeal host cell [48] (see “Methods” section). Each classifier yields a “viralness” score, which can be used to determine the likelihood of the input sequence to represent a partial or complete genome from the corresponding viral group.

**Table 1:** Features used in Virsorter2, VirSorter, and MARVEL. Detailed explanation of each feature is provided in the “Methods” section. In VirSorter, features 3 to 6 are summed up as one feature (% of Pfam affiliated genes)

	VirSorter2	VirSorter	MARVEL
1. Hallmark gene count	x	x	
2. % of viral genes	x	x	x
3. % of archaeal genes	x	x	
4. % of bacterial genes	x		
5. % of eukaryotic genes	x		
6. % of mixed genes	x		
7. % of unaligned genes	x	x	
8. Average gene size	x	x	x
9. Gene overlapping frequency	x		
10. Gene density	x	x	x
11. Strand switching frequency	x	x	x
12. % of ATG as starting codon	x		x
13. % of GTG as starting codon	x		
14. % of TTG as starting codon	x		
15. Mean of GC content	x		
16. SD of GC content	x		
17–27. % of RBS motifs	x

To train the different classifiers, reference genomes for each viral group were split into training and testing sets with a 9 to 1 ratio, respectively, and 5 random fragments were generated from each genome starting at a random position and with size ranging from 1 kb to complete genomes. Separate classifiers were trained for the following viral groups (i) caudovirids (*Caudovirales*) and comparable dsDNA phages (e.g., corticovirids), (ii) NCLDVs, (iii) RNA viruses, (iv) ssDNA viruses, and (v) lavivirids (*Lavidaviridae*). Separately, a negative (non-viral) dataset was generated from genomes of three groups: (i) bacteria and archaea, (ii) eukaryotes (fungi and protozoa), and (iii) plasmids. The training and testing sets for non-viral genomes were generated the same way as the viral set described above.

For each input sequence to be classified, all random forest classifiers are first applied to the entire sequence. If the score obtained with one or more of the classifiers is above the cutoff set by the user (the default was 0.5, as used in the classifier training step), the score is considered significant and the sequence was considered as entirely or near-entirely viral. To identify potential host regions on the edge of these contigs, sub-sequences of the input sequence with 0 to 5 genes or 10% of total genes (whichever larger) trimmed in 5′ and/or 3′ processed with the same classifiers, and selected if the viralness score increased compared to the full sequence. If no score was significant when analyzing the complete sequence, the same classifiers are applied to sliding windows across the input sequence. The size of the sliding window was determined separately for each classifier as the minimal size of a genome from the corresponding viral group (Table S1). The sliding window starts from the 5′ edge of a contig and shifts one gene at a time while there is no significant score. For each window yielding a significant score, this window is extended one CDS at a time in 3′ as long as the score stays significant. Eventually, overlapping regions identified by different classifiers are compared and the longest prediction is retained.

### Expanded and manually curated databases enable a robust detection of viruses in the order *Caudovirales*

We first evaluated the new VirSorter2 approach on genomes in the order *Caudovirales*. As described above, test sequences included both phage isolate sequences from NCBI Viral RefSeq [49] and uncultivated phage genomes obtained from metagenomes or identified as proviruses in bacterial and archaeal whole genome shotgun sequencing ([15, 16, 48], see “Methods” section). Test sequences also did not overlap with the references used to train the classifier. As negative control, the same number of sequences from the non-viral testing set were included. The same test sequences were also processed with established viral sequence detection tools including VirSorter [29], VirFinder [33], DeepVirFinder [34], MARVEL [31], and VIBRANT [32]. The overall performance of each tool was evaluated using the F1 score, which represents the harmonic mean of precision and recall [50].

**Fig. 3:**
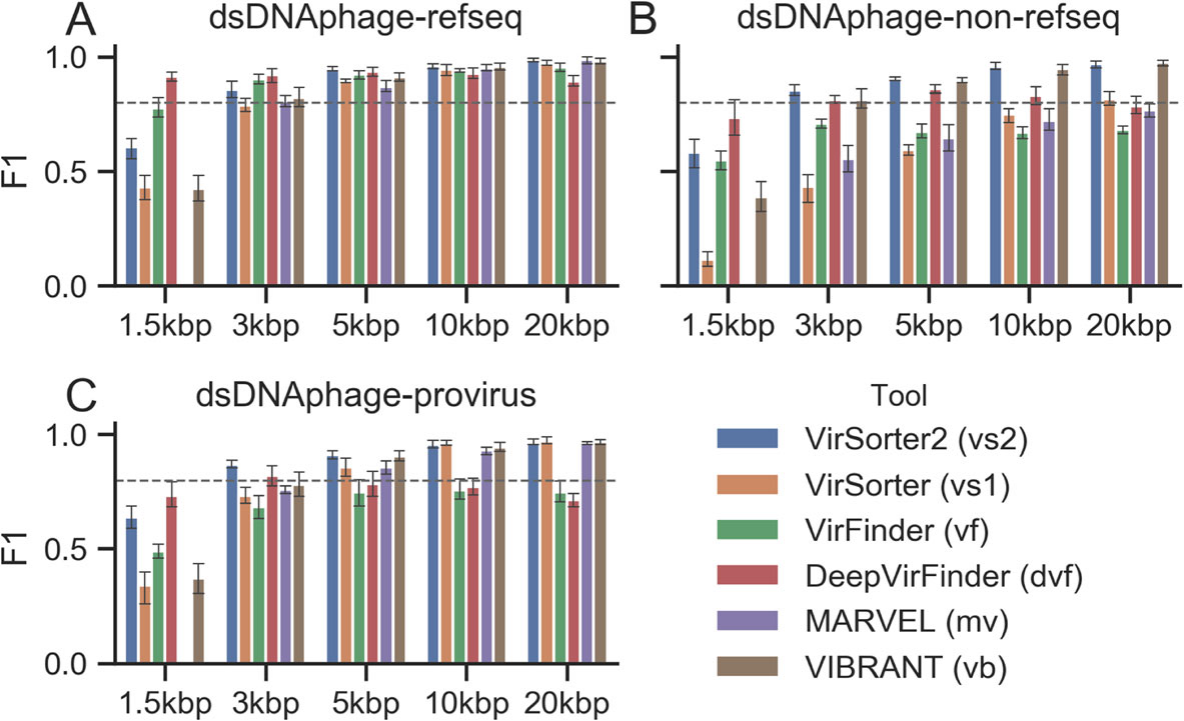
Tool performances on dsDNA phages from different data sources. VirSorter2 consistently has comparable or better performance than existing tools in identifying dsDNA phages. Genome fragments of different lengths (*x*-axis) are generated from genomes in the order *Caudovirales* in NCBI Viral RefSeq (**a**), proviruses extracted from microbial genomes in NCBI RefSeq (**b**) [48], and other sources (**c**) [15, 16]. An equal number (50) of viral and non-viral (archaea and bacteria, fungi and protozoa, and plasmids) genome fragments were combined as an input for the tested tools. Error bars show 95% confidence intervals over five replicates (100 sequences each as described above). F1 score is used as the metric (*y*-axis) to compare tools, while detailed recall and precision results are available in Figs. S1 and S2. The dotted line is *y* = 0.8

Overall, all tools performed well in the identification of viral sequences in the order *Caudovirales* from RefSeq, as they all displayed F-score ≥ 0.8 with sequences 5 kb and longer (Fig. 3a, Figs. S1 & S2). As previously observed [29, 39], all approaches also displayed a decreased accuracy with shorter sequences, especially when reaching lengths of ~ 1–2 kb (Fig. 3a). While increased accuracy with sequence length was also observed when evaluating the same tools with uncultivated viral sequences of the order *Caudovirales*, the overall performance of several tools was lower (Fig. 3b, c, Figs. S1 & S2). Specifically, the performance of VirSorter, VirFinder, DeepVirFinder, and MARVEL was decreased by 10–30%, while VIBRANT and VirSorter2 displayed F-score comparable to those when evaluated with the RefSeq sequences (± 5%), likely due to their larger reference database and their ability to use hallmark genes to identify viruses only distantly related to RefSeq genomes. Similar results were obtained when analyzing viral sequences, either from RefSeq or uncultivated, for which < 25% of the genes were annotated as viral based on the custom HMM database (Fig. S3), confirming that the differences in tool performances were mostly due to the ability to identify novel viruses. To verify that the VirSorter2 approach was similarly efficient for integrated proviruses, we processed bacterial genomes from a gold-standard set of 51 microbial genomes [51] for which 82 integrated proviruses have been manually curated. As for viral contigs, VirSorter2 recall and precision was comparable or higher to all other tools (Table 2). Overall, the approach used by VirSorter2 to identify *Caudovirales* is thus on par or more efficient than other recently published tools for both isolate and environmental sequences, across a broad range of contig length, and for sequences both entirely and partially viral. The only exception was for viral contigs of < 3 kb that have representation in NCBI RefSeq, which were better recovered by kmer-based methods such as VirFinder and DeepVirFinder (Fig. 3a).

**Table 2:** Performance of VirSorter2 and previously published prophage identification tools when calling proviruses from the standard dataset described in Casjens [51]

	PHAST (all categories)	PHAST (no questionable)	PhiSpy	Phage Finder	VirSorter (categories 1 and 2)	Virsorter (ll categories)	VirSorter2
Recall	84.27%	70.04%	78.28%	64.42%	73.41%	79.30%	94.38%
Precision	83.03%	82.74%	73.59%	79.26%	92.89%	72.24%	73.04%

### Dedicated custom models allow identification of diverse viral sequences

We next reasoned that, since different viral groups have different defining characteristics, a single model was unlikely to efficiently handle the entire viral diversity. Hence, just as viral taxonomists have to use different characteristics to classify viruses across the known virosphere [52], different rules would be needed to robustly identify different types of viruses in nature instead of the single model used by all other tools available to date, including the original VirSorter. To demonstrate the effectiveness of this multi-classifier approach, we focused our efforts on four major virus groups outside the order *Caudovirales* where significant data and expertise have accumulated—ssDNA viruses, RNA viruses, NCLDV, and lavidavirids (virophages)—as described above.

**Fig. 4:**
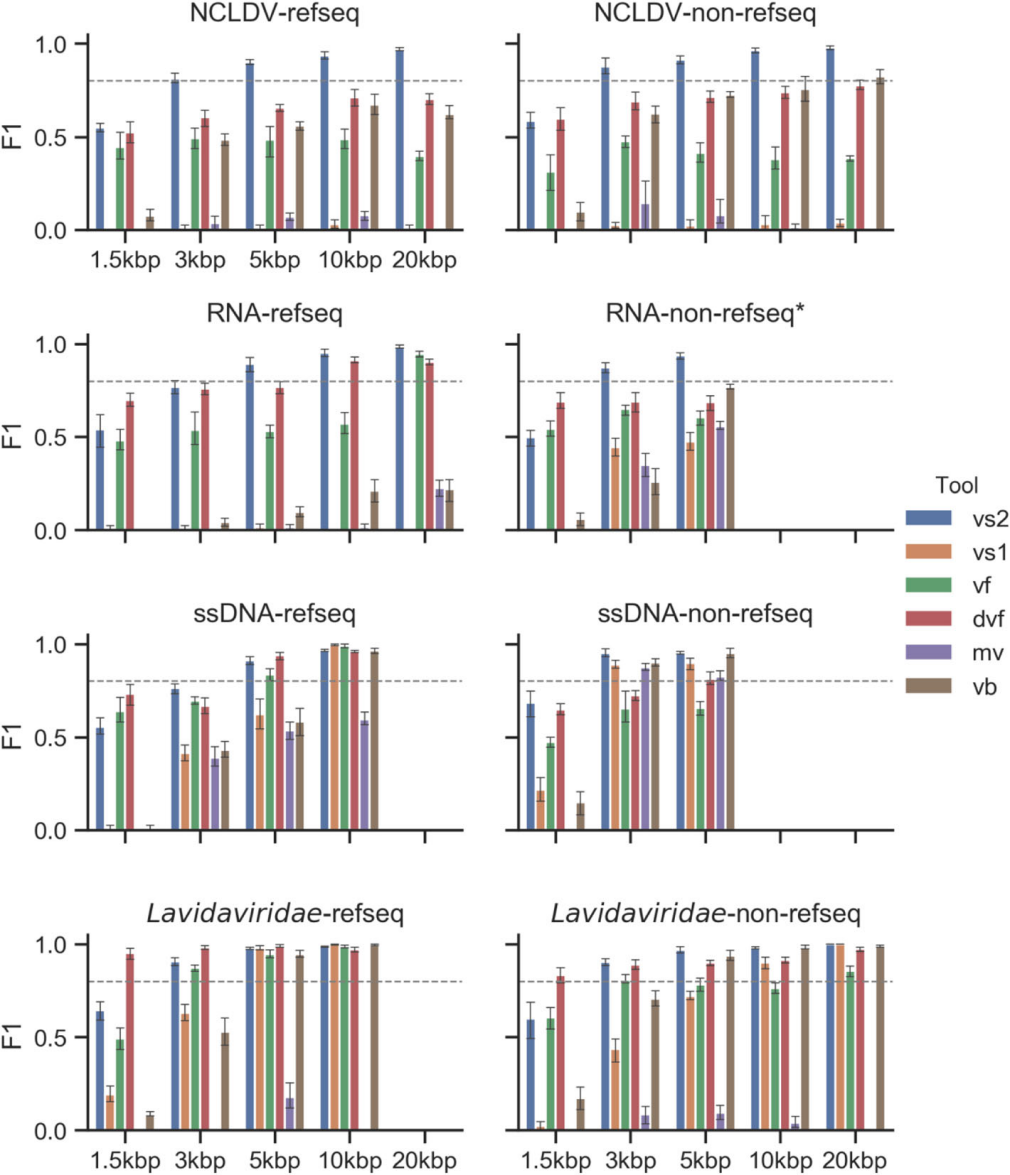
Tool performances on different viral groups (other than dsDNA phage) from different data sources. VirSorter2 consistently outperforms existing tools in identifying viral groups outside dsDNA phages Genome fragments of different lengths (*x*-axis) are generated from NCBI RefSeq (“refseq”) genomes in each viral group and other sources (“non-refseq”) [19, 45–47, 53]. “RNA-non-refseq*” is a collection of ssRNA phage genomes [45]. An equal number (50) of viral and non-viral (archaea and bacteria, fungi and protozoa, and plasmids) genome fragments were combined as an input for the tested tools. F1 score is used as the metric (*y*-axis) to compare tools. The dotted horizontal line is *y* = 0.8. *vs2* VirSorter2, *vs1* VirSorter, *vf* VirFinder, *dvf* DeepVirFinder, *mv* MARVEL, *vb* VIBRANT

Comparing the range and median value of each feature between groups confirmed that features such as gene density or percentage of viral gene could clearly differ between groups (Fig. 2). The relative importance of each feature in individual classifiers was also variable between different groups, confirming fundamental differences in genome organization and content between viruses (Fig. S4). Comparing the performance of VirSorter2 to other existing approaches on these non-*Caudovirales* viruses demonstrated that most of the tools evaluated were not able to consistently identify these sequences (Fig. 4, Figs. S5 & S6). Overall, current virus detection tools typically struggled with at least one type of non-*Caudovirales* virus, and also showed reduced performance for sequences not currently part of NCBI Viral RefSeq, i.e., more distantly related to known references. Meanwhile, VirSorter2 was the only tool which displayed F1-scores > 0.8 across all groups for contigs 5 kb and longer, and always had the highest F-score for all contigs ≥ 3 kb except for RNA viruses from RefSeq where DeepVirfinder performed better. Through its modular framework, VirSorter2 is thus uniquely able to reliably detect different types of non-*Caudovirales* viruses. Importantly however, VirSorter2’s F1-score substantially decreased for sequences < 3 kb, as previously observed for the viruses in the order *Caudovirales*, due to loss of sensitivity (Figs. S5 and S6). Thus, the VirSorter2 approach is currently not optimal for short (< 3 kb) contigs.

The decrease in F1-score between VirSorter2 and the next best tool varied between groups and lengths, from 4.4% for the ssDNA to 31.8% for NCLDV on average with sequences longer than 5 kb, highlighting that some groups benefited from a separate classifier more than others (Fig. 4, Figs. S5 & S6). Pragmatically, this means that lavidavirid gene content and genome structure may be close enough to the “extended *Caudovirales*” group that it could potentially be included in the same classifier, whereas NCLDV and ssDNA viruses are better identified when considered separately. VirSorter2 was thus designed modularly to enable modification and addition of new classifiers as our knowledge of viral sequence space increases.

### Plasmids and eukaryotic genomes represent unique challenges for viral detection tools

**Fig. 5:**
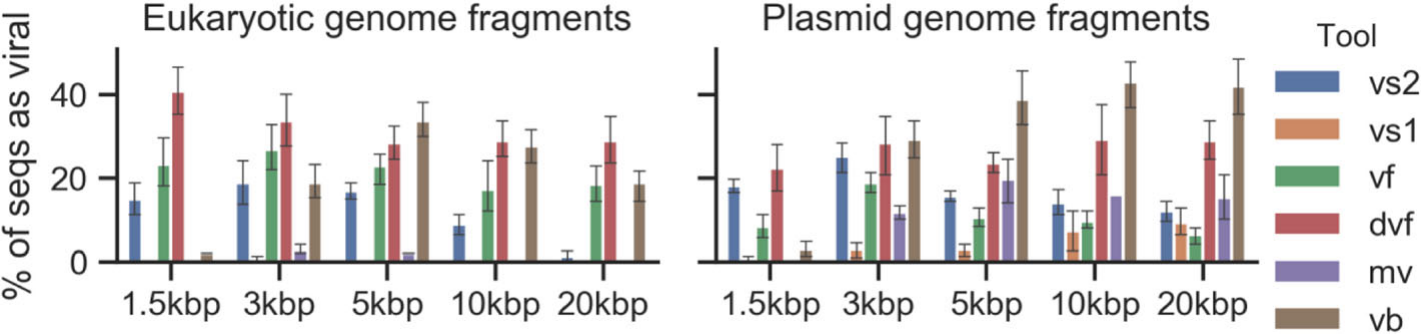
False positives comparison of tools on eukaryotes and plasmids. Genome fragments (50) of different lengths (*x*-axis) were generated from eukaryotic genomes (fungi and protozoa) in NCBI RefSeq, and plasmids. Percent of genome fragments classified as viral is used as the metric (*y*-axis) to compare tools. *vs2* VirSorter2, *vs1* VirSorter, *vf* VirFinder, *dvf* DeepVirFinder, *mv* MARVEL, *vb* VIBRANT

While initial tests were performed using random fragments of bacterial and archaeal genomes as non-virus sequences, previous studies showed that eukaryotic genome and plasmid fragments were especially prone to be mis-identified as a putative viral sequence [39]. We thus evaluated how VirSorter2 and other tools handled both eukaryotic and plasmid sequences, by processing datasets entirely composed of eukaryotic genome fragments or plasmid fragments and measuring at which frequency these would be wrongly considered as viral (Fig. 5).

For eukaryotic genomes, both MARVEL and VirSorter showed high specificity with 2.4% and 0.1% false positives on average respectively, while VirSorter2 and VIBRANT displayed on average 12.1% and 20.9% of false positives respectively (Fig. 5a). The error rate was consistent across sequence length for all tools except for VirSorter2, where this error rate decreased quickly when sequence length increased, to reach < 1% for fragments ≥ 20 kb. These differences in specificity are consistent with the original scope of the tools: both VirSorter and MARVEL were designed to identify bacteriophages and archaeoviruses [29]. While they do not mistake eukaryotic sequences as viral, they also poorly detect eukaryotic viruses such as NCLDV (Fig. 4). VirSorter2 and VIBRANT are both able to recover eukaryotic virus sequences, such as NCLDV, and RNA viruses, but do so at the cost of a specificity loss that is relatively similar across the two tools. However, VirSorter2’s modular framework can be leveraged by users interested in particular subsets of viruses to reduce these losses. For example, to target viruses of bacteria and archaea, users can omit the NCLDV, *Lavidaviridae*, and RNA virus classifiers to drop VirSorter2 error rate associated with eukaryotic genomes, as demonstrated by a reduction in false-positive rate to < 1.5% for all fragment lengths on average (Fig. S7). Finally, both VirFinder and DeepVirFinder were most likely to mistake eukaryotic sequences as viruses as they had more than double the false positive rate of VirSorter2 (21.7% for VirFinder, 32.1% for DeepVirFinder on average). This high eukaryote-for-virus error rate for k-mer-based methods, including for large (20 kb) sequences, is a known limitation of these approaches [39].

In contrast to eukaryotic genome sequences, no tool performed well against plasmid sequences (Fig. 5b). This included both gene-content and k-mer based tools, and stayed true even for large plasmid fragments (e.g., ≥ 20 kb) for which the error rate ranged between 6.4 and 42.0% for all tools. This highlights how plasmid sequences cannot be entirely distinguished from viruses using current approaches and suggests that alternative methods need to be designed for this purpose. Given the growing list of examples of how plasmid and viral sequence space are intertwined [54–57], significant work in this area will presumably be required, including possibly the incorporation of a dedicated tool for plasmid detection in VirSorter2 modular framework.

### VirSorter2 is easily available and can be scaled to handle large-scale datasets

Beyond accurate and sensitive virus identification, scalability to process large (> 100,000 sequences) datasets and broad availability for users regardless of their knowledge of command-line or scripting are desirable traits in virus sequence detection tools. For the former, VirSorter2 can process an input dataset of 100,000 sequences totaling 4.2 Gb in 28.6 h using 32 threads. Its memory usage stays nearly constant with increasing data size, and scales nearly linearly with threads used (Figs. S8 & S9). Its total CPU time is higher than other tools (typically 2–10× higher, Fig. S8), but VirSorter2’s internal processes are highly parallelizable, and written with snakemake, a cutting-edge pipeline management tool designed for computer clusters [58]. VirSorter2 is thus able to utilize multiple cores in the same node and across multiple nodes efficiently, so that compute time would not be a limiting factor even for (ultra)large-scale datasets if state-of-the-art computing cluster resources are available (Fig. S9). Further, this increased CPU time is mostly due to the annotation step with larger viral HMM database (> 90% of CPU time) and not to the use of several distinct classifiers, suggesting that additional viral groups and classifiers could be added to the VirSorter2 framework with minimal impact on performance. Finally, users can explore the annotation and per-model score computed by VirSorter2 for each individual sequence, and can generate new output files using different options and cutoffs based on an existing VirSorter2 directory without having to re-compute the annotation step. To illustrate this and other differences between VirSorter and VirSorter2, we provide a detailed analysis of viral sequence detection from an ocean virome using both VirSorter and VirSorter2 (Supplementary Text, Fig. S10, Additional files 4 and 5).

In order to make VirSorter2 useful for the largest community possible, we provide it through multiple implementations and accompanied with extended documentation. Specifically, VirSorter2 is freely available at bitbucket (https://bitbucket.org/MAVERICLab/VirSorter2), as an App on the CyVerse (https://de.cyverse.org/de), and available for local installation through a bioconda package (https://anaconda.org/bioconda/virsorter). In addition, we provide an extensive step-by-step tutorial on how to run VirSorter2 and how to design new classifiers for additional viral groups and integrate this within a local VirSorter2 instance on protocols.io/VERVENET (https://www.protocols.io/view/getting-started-with-virsorter2-bhdij24e).

### Current limitations and future developments

Together, these benchmark experiments demonstrate that by integrating multiple classifiers, each customized for a specific viral group, VirSorter2 vastly improves the diversity of viruses that can be automatically detected from environmental sequence data. However, we note several limitations that will benefit from future improvements in our understanding of the virosphere and technical capabilities.

First, while separating the global viral diversity in different groups clearly improved viral sequence detection, there is currently no automated process to optimally group reference viral sequences into different viral classifiers. If the viral groups selected are too large and/or diverse, the corresponding classifier would likely suffer from the same flaw as previous tools, whereby rare members would be under-detected. Conversely, classifiers designed for groups lacking diverse representation will show reduced accuracy, due to an under-trained random forest classifier being unable to extract meaningful identifying features for the group. Thus, further virosphere exploration and systematic classification is critical to provide the training data needed to optimize detection.

Second, some viral groups may require additional references and/or features to achieve a systematic and robust identification. The limits associated with reference sequences are shared by all viral sequence identification tools: as new viral genomes and groups are discovered, e.g., high-quality genomes assembled from metagenomes [59], these need to be integrated into virus detection approach to keep these up-to-date. This iterative process is now facilitated by the modular framework of VirSorter2, for which we provide a detailed step-by-step protocol to add additional viral groups and classifiers. The addition of other features would also be a possible avenue for extending the range and/or accuracy of VirSorter2. Contrary to the addition of new viral groups, integrating new feature(s) would require substantial modification of VirSorter2 code, and would thus be only associated with the release of new versions of the tool.

Finally, while run times and computational resources required by VirSorter2 are compatible with modern omics datasets, the size of these datasets will likely keep increasing over the next few years, and may eventually limit the usefulness of VirSorter2. Given that most of the computation time is currently dedicated to the annotation step, this issue will require integration of scalable and improved sequence comparison tools (e.g., MMSeqs2 [60], HHBlits [61]) and/or new versions of the tools currently used.

## Conclusion

The automatic extraction of viral sequences from large-scale sequencing data is now a cornerstone of the current viral ecogenomics toolkit. Identifying viral genomes from omics data enables unprecedented studies of viral taxonomic diversity [62], viral population distribution and associated eco-evolutionary constraints [15, 16, 47], and viral potential for microbial metabolic reprogramming [20]. While current tools typically detect known and “standard” viruses, i.e., viruses in the order *Caudovirales*, pretty well, the approaches available to date remain challenged by novel and/or unusual viral groups. VirSorter2 now provides a framework to go beyond the detection of viruses in the order *Caudovirales* and enables robust detection of all types of viruses in sequencing data. By defining subgroups of viruses with consistent genome features and unique markers, the VirSorter2 framework is designed to grow as databases do such that new viral diversity can be readily detected in large-scale datasets. This will in turn enable researchers to investigate the role(s) played by all viruses across Earth’s biomes, and better understand how these viruses constrain fundamental microbial processes.

## Methods

### Viral HMM database

The viral HMM database includes viral HMMs from viral protein families (VPF) of JGI earth’s virome project [17] and Xfams (https://de.cyverse.org/dl/d/6489360D-1126-413B-A667-D18E39D5F2F1/viral_db_default.hmm). Xfams were generated from a large collection of viral sequences from the Global Ocean Viromes 2.0 (GOV 2.0) [16] and the Stordalen Mire Viromes (SMV) [63]. Briefly, viral contigs were identified by VirSorter [29], DeepVirFinder [34], and MARVEL [31]. The intersection between the three tools (Category 1 in VirSorter with “--virome” mode, score ≥ 90% in Marvel, and a score of ≥ 0.9 with a *p* value of < 0.05 in DeepVirFinder), were kept. Open reading frames (ORF) were predicted using prodigal with “--meta” mode [41], filtered by removing ones with ≥ 95% similarity to RefSeq’s bacterial and archaeal proteins, and then clustered by ClusterONE [64] to get rid of singletons (-d 0.3 -s 0.2 --max-overlap 0.8). Multiple sequence alignments were generated for each cluster by MUSCLE (--maxiters 4) [65], and then made into HMMs by hmmbuild in HMMER3 package [66]. More details of Xfams can be found at iVirus in CyVerse’s Discovery Environment (https://de.cyverse.org/de/): /iplant/home/shared/iVirus/Xfams/version_0.5/Xfams-XC.

### Training classifiers

NCBI RefSeq genomes including archaea, bacteria, protists, fungi, and virus were downloaded from NCBI (ftp://ftp.ncbi.nlm.nih.gov/genomes/refseq) on 2020-01-12. Viral genomes from the order *Caudovirales* were used for dsDNA phage; genomes from the families *Mimiviridae*, *Phycodnaviridae*, *Faustoviridae*, *Iridoviridae*, *Marseilleviridae*, *Ascoviridae*, *Pithoviridae*, *Poxviridae*, and *Pandoraviridae* were used for NCLDV; genomes from the realm *Riboviria* were used for RNA viruses; genomes from the families *Bacilladnaviridae*, *Circoviridae*, *Geminiviridae*, *Nanoviridae Genomoviridae*, *Parvoviridae*, *Microviridae*, *Smacoviridae*, *Alphasatellitidae*, *Tolecusatellitidae*, *Anelloviridae*, *Bidnaviridae*, *Pleolipoviridae*, *Spiraviridae*, and *Inoviridae* were used for ssDNA viruses; genomes from the family *Lavidaviridae* were used for *Lavidaviridae* classifier. Other than NCBI RefSeq genomes, a set of high-quality genomes was also obtained from the literature [15, 16, 19, 45–47, 53] and unpublished ssDNA virus genome data (Varsani, unpublished data). These genomes were identified based on VirSorter1, VirFinder, or using custom pipelines, and were further analyzed and manually curated as part of their respective publication. Hence, they represent useful examples of “novel” viruses, often distantly related to RefSeq reference genomes. The genomes of all viral and non-viral groups were split into “training” and “testing” sets with a 9 to 1 ratio, respectively. The “training” sets from RefSeq and non-RefSeq sources were combined. For viral genomes, five fragments were generated starting at a random genome position with length ranging from 1000 bp to the maximal length (extending to the end of genome). For bacteria, archaea, and eukaryotes, five fragments were generated from a representative genome from each genus in the same way as described above; for plasmids, because no systematic global classification is available and the total number of plasmids in the input dataset [67] was only 6642 sequences, five fragments were generated from each sequence. The generated fragments from each of the three non-viral groups (bacteria and archaea, eukaryotes, and plasmids) were subsampled to the minimum within these three negative groups (bacterial and archaea, eukaryotes, and plasmids) and combined as a final “negative set.” For each viral group, the viral “training set” and the non-viral “training set” were evenly subsampled to the smaller size between the two, and then used for training the classifier. Then the following 27 features were extracted from each sequence fragment:

1. “Hallmark gene count” is the count of hallmark genes in a viral sequence
2. “% of viral genes” is calculated as the percent of genes annotated as viral (best hit to viral HMMs) of all genes
3. “% of archaeal genes” is calculated as the percent of genes annotated as archaeal (best hit to archaeal HMMs) of all genes
4. “% of bacterial genes” is calculated as the percent of genes annotated as bacterial (best hit to bacterial HMMs) of all genes
5. “% of eukaryotic genes” is calculated as the percent of genes annotated as eukaryotic (best hit to eukaryotic HMMs) of all genes
6. “% of mixed genes” is calculated as the percent of genes with best hit to HMMs shared between viruses and cells
7. “% of unaligned genes” is calculated as the percent of genes with no hits to HMMs in the VirSorter2 HMM database (bit score cutoff 30)
8. “Average gene size” is the mean of gene sizes in a viral sequence
9. “Gene overlapping frequency” is the percent of consecutive genes that are on the same strand and overlap when scanning from 5′ to 3′ on the + strand
10. “Gene density” is the average number of genes in every 1000 bp sequence window (total number of genes divided by contig length and then multiplied by 1000)
11. “Strand switching frequency” is the percent of genes located on the opposite strand from the gene upstream (scanning from 5′ to 3′ in the + strand)
12. “% of ATG as starting codon” is the percent of gene with ATG as starting codon
13. “% of GTG as starting codon” is the percent of gene with ATG as starting codon
14. “% of TTG as starting codon” is the percent of gene with ATG as starting codon
15. “Mean of GC content” is the mean of GC content of all genes in a sequence
16. “SD of GC content” is the standard deviation of GC content of all genes in a sequence
17. “% of RBS motifs” is percent of ribosomal binding sites (RBS) with a specific motif. There are 11 types of motifs included: SD_Canonical, SD_Bacteroidetes, TATATA_3-6, OnlyA, OnlyT, DoubleA, DoubleT, Other_GA, NoA, Other, and None (no motif found) [19].

Features 8–27 are all extracted from the gff output from prodigal [41].

With the above features, “RandomForestClassifier” in scikit-learn package [50] was used to train the random forest classifier. “MinMax” scaler was used to scale all the feature data, and “GridSearchCV” was used to find optimal parameter sets among “n_estimators” of 20, 50, 100, 150, 200, and “criterion” of gini or entropy.

### Accuracy

To compare the accuracy of VirSorter2 to other viral identification tools, datasets with equal number (50) of viral and non-viral random DNA fragments were generated from the testing set created above and with different lengths (1.5, 3, 5, 10, and 20 kb) to discern the impact of sequence length on accuracy. VirSorter2 (version 2.0.beta) was run with “--include-groups dsDNAphage, NCLDV, RNA, ssDNA, lavidaviridae, --min-score 0.5,” and sequences in “final-viral-combined.fa” were considered as viral. VirSorter (version 1.1.0) was run with “--db 2 --virome –diamond,” and sequences in categories 1, 2, 4 and 5 were considered as viral; VIBRANT (version 1.2.1) was run with “--virome -f nucl”, and sequences in “VIBRANT_*/VIBRANT_phages_*/*.phages_combined.fna” were considered as viral; VirFinder (version 1.1) was run with VF.pred function within R as described in (https://github.com/jessieren/VirFinder); DeepVirFinder was installed directly from GitHub (https://github.com/jessieren/DeepVirFinder) with last commit ID of ddb4a9433132febe5cda39548cb9332669e11427 and was run with default parameters; for both VirFinder and DeepVirFinder, sequences with p value < 0.05 were chosen as viral. Since MARVEL (version 0.2) required each viral genome as a separate file, input sequences were first split so that each sequence was an individual sequence file, and then default parameters were used, and the sequences in “results/phage_bins” were considered as viral. F1 score (harmonic mean of recall and precision) was used as a metric for tool accuracy comparisons. For measuring false positives on eukaryotic and plasmid sequences, the fraction of eukaryotic or plasmid sequences classified as viral were calculated. To test performance on provirus detection, 54 bacterial genomes with known provirus boundary [51] were used. VirSorter2 was run with the “--include-groups all, --min-score 0.5” flag. If a provirus has ≥ 50% of its genome recovered, it was considered as a true positive, and the VirSorter2 identified viral sequences without any true positives identified in them were considered as false positives. Recall and precision of other tools [28, 68, 69] were retrieved from the original VirSorter benchmarking experiments [29].

### Computational efficiency

To measure how tools scale with input data size, datasets with 10, 100, and 1000 sequences of 10 kb in length were generated in the same way as in accuracy benchmarking described above. To measure multithreading efficiency, datasets with 1000 sequences of 10 kb were generated and used as an input for VirSorter2, VirSorter, MARVEL, and VIBRANT were run with 1, 2, 4, 8, 16, 32 threads. VirFinder was not included since it did not have a multi-threading option. DeepVirFinder had unexpected behavior with its multi-threading option (“-c”), i.e., the “-c” option could not control thread number, so was also not included. CPU time and run time was measured with “/usr/bin/time -v” command available in the Linux operating system and peak memory was measure with “memusg” script (https://gist.github.com/netj/526585).

## Supplementary Information

**Additional file 1: Figure S1.** Recall comparisons of tools on dsDNA phages from different data sources. VirSorter2 consistently has comparable or better performance than existing tools in identifying dsDNA phages. Genome fragments of different lengths (x-axis) are generated from genomes in the order *Caudovirales* in NCBI Viral RefSeq (A), proviruses extracted from microbial genomes in NCBI RefSeq (B) and other sources (C) (described in the “Training classifier” part of the Method section). An equal number (50) of viral and non-viral (archaea and bacteria, fungi and protozoa, and plasmids) genome fragments were combined as an input for the tested tools. Error bars show 95% confidence intervals over five replicates (100 sequences each as described above). Recall is used as the metric (y-axis) to compare tools. The dotted line is y = 0.8. **Figure S2.** Precision comparisons of tools on dsDNA phages from different data sources. Genome fragments of different lengths (x-axis) are generated from genomes in the order *Caudovirales* in NCBI Viral RefSeq (A), proviruses extracted from microbial genomes in NCBI RefSeq (B) and other sources (C) (described in the “Training classifier” part of the Method section). An equal number (50) of viral and non-viral (archaea and bacteria, fungi and protozoa, and plasmids) genome fragments were combined as an input for the tested tools. Error bars show 95% confidence intervals over five replicates (100 sequences each as described above). Precision is used as the metric (y-axis) to compare tools. The dotted line is y = 0.8. **Figure S3.** Tool performances with viral sequences having < 25% of the genes annotated as viral. Genome fragments of different lengths (x-axis) were generated from *Caudovirales* genomes from both NCBI RefSeq genomes and other sources. Only data sources with > 10 viral sequences that have < 25% genes annotated as viral were kept. Then equal numbers (50) of viral and non-viral (archaea and bacteria, fungi and protozoa, and plasmids) genome fragments were combined as an input for the tested tools. F1 score is used as the metric (y-axis) to compare tools. vs2 = VirSorter2; vs1 = VirSorter; vf = VirFinder; dvf = DeepVirFinder; mv = MARVEL; vb = VIBRANT. **Figure S4.** Importance of different features for viral sequence identification across viral groups. The y-axis shows the relative contribution of individual features in separating the training viral and nonviral (bacterial and archaea, fungi and protozoa, and plasmid) data (total is 1), provided by the Random Forest classifier after processing training data, and based on the F1 score. Top four features from each viral group (10 in total) are shown. In the features (color), “vir” (% of viral genes) is calculated as the percent of genes annotated as viral (best hit to viral HMMs) of all genes; “bac” (% of bacterial genes) is calculated as the percent of genes annotated as bacterial (best hit to bacterial HMMs) of all genes; “hallmark” (hallmark gene count) is the count of hallmark genes in a viral sequence; “mix” (% of mixed genes) is calculated as the percent of genes with best hit to HMMs not specific to virus or non-virus; “Strand_switch_perc” (Strand switching frequency) is the percent of genes located on a different strand from the previous gene (scanning from 5′ to 3′ in the + strand); “density” (Gene density) is the average number of genes in every 1000 bp sequence (total number of genes divided by contig length and then multiply by 1000); “gc_mean” (Mean GC content) is the mean of GC content of all genes in a contig; “atg_perc” (% of ATG as start codon) is the percent of genes with ATG as a starting codon; “rbs_None” is the percent of ribosomal binding sites (RBS) with no motif detected; “rbs_TATATA_3-6” is the percent of RBS with “TATATA_3-6” motif. **Figure S5.** Recall comparisons of tools on different viral groups (other than dsDNA phage) from different data sources. Genome fragments of different lengths (x-axis) are generated from NCBI RefSeq (“refseq”) genomes in each viral group and other sources (“non-refseq”). Then equal numbers (50) of viral and non-viral (archaea and bacteria, fungi and protozoa, and plasmids) genome fragments were combined as input for tools. Recall was used as the metric (y-axis) to compare tools. The dotted horizontal line is y = 0.8. vs2 = VirSorter2; vs1 = VirSorter; vf = VirFinder; dvf = DeepVirFinder; mv = MARVEL; vb = VIBRANT. **Figure S6.** Precision comparisons of tools on different viral groups (other than dsDNA phage) from different data sources. Genome fragments of different lengths (x-axis) are generated from NCBI RefSeq (“refseq”) genomes in each viral group and other sources (“non-refseq”). Then equal numbers (50) of viral and non-viral (archaea and bacteria, fungi and protozoa, and plasmids) genome fragments were combined as input for tools. Precision was used as the metric (y-axis) to compare tools. The dotted horizontal line is y = 0.8. vs2 = VirSorter2; vs1 = VirSorter; vf = VirFinder; dvf = DeepVirFinder; mv = MARVEL; vb = VIBRANT. **Figure S7.** False positives by VirSorter2 on eukaryotes and plasmids. Genome fragments (50) of different lengths (x-axis) are generated from eukaryotic genomes (fungi and protozoa) in NCBI RefSeq, and plasmids. Percent of genome fragments classified as viral was used as the metric (y-axis) to evaluate tools. Plot A and C show contribution of each classifier (color) to total false positives in VirSorter2 (as shown in Fig. 4) for eukaryotes and plasmid respectively. Plot B and D show the total false positive in VirSorter2 after excluding NCLDV, RNA, and *Lavidaviridae* classifiers. vs2 = VirSorter2. **Figure S8.** CPU time and peak memory comparison among tools across data sizes. Tools were run on different input sizes of 10, 100, 1000 sequences with 10 kb in length. Plot (A) shows all tools scale nearly linearly with data size, and (B) shows peak memory usage of all tools are <1 GB. VirSorter2 and VirFinder peak memory usage stay nearly constant. vs2 = VirSorter2; vs1 = VirSorter; vf = VirFinder; dvf = DeepVirFinder; mv = MARVEL; vb = VIBRANT. **Figure S9.** Multi-threading efficiency comparison among tools. Tools were run on 1000 sequences with 10 kb in length. Plot (A) shows VirSorter2 and VIBRANT have the best multi-threading efficiency, i.e. total run time decreases nearly linearly with the number of threads used. VirSorter can not use more than four threads. MARVEL’s multi-threading option does not significantly reduce run time. Plot (B) shows VirSorter2, VirSorter and VIBRANT memory usage increases with the number of threads used, with VirSorter2 and VIBRANT increasing at a higher rate than VirSorter. MARVEL’s memory usage stayed constant. vs2 = VirSorter2; vs1 = VirSorter; mv = MARVEL; vb = VIBRANT. **Figure S10.** Overview of VirSorter2 results for *Tara* Oceans virome 85_SRF. A. Detection of viral contigs via VirSorter 1.0.4 and VirSorter 2.0.beta by contig size. The top panel displays the total number of viral contigs identified in each size class, while the bottom panel indicates the overlap between these predictions. B. Distribution of VirSorter 2.0.beta score (maximum score across all 5 classifiers, y-axis) for *Tara* Oceans virome 85_SRF sequences, according to the confidence category estimated by VirSorter1 (x-axis). “NA” indicates contigs that were not detected as viral by VirSorter1. VirSorter 2.0.beta detections were based on a minimum score cutoff of 0.5. C. Proportion of sequences from Tara Oceans virome 85_SRF detected as viral based on the dsDNAphage, RNA, and/or ssDNA model(s) (red) or detected based on the NCLDV or *Lavidaviridae* classifiers only, by size class (x-axis). **Table S1.** Summary statistics of the genomes used for training VirSorter2 classifiers of different viral groups from RefSeq and non-RefSeq sources. “Genome #” is the number of genomes. “Min size”, “Median size”, and “Max size” are the minimum, median, and maximum of genome sizes per each viral group.

**Additional file 2. **Case-study: identifying viral contig from a Tara Oceans virome dataset

**Additional file 3.** First 20 rows of the “final-viral-score.tsv” VirSorter2 output file for Tara Oceans virome 85_SRF. VirSorter 2.0.beta was used with default parameters, including all classifiers and a minimum score cutoff of 0.5. The columns include first the sequence name, the score for each of the 5 classifiers, the maximum score for this sequence, the group yielding this maximum score, the sequence length, the number of hallmark gene(s) for the maximum score group, and the percentage of viral and cellular genes.

**Additional file 4.** Manual inspection of contigs newly identified by VirSorter2 in Tara Oceans virome 85_SRF. The spreadsheet includes four tabs, including the list and characteristics of contigs ≥ 5kb selected for manual inspection (“Contigs ≥ 5kb - Manual inspection”), the DRAM-v annotation of these contigs (“Contigs ≥ 5kb - DRAM-v annotation”), the list and characteristics of contigs ≥ 5kb selected for manual inspection (“Contigs < 5kb - Manual inspection”), and their DRAM-v annotation (“Contigs < 5kb - DRAM-v annotation”). For the manual inspection tabs, column headers correspond to the standard output of VirSorter2 (“final-viral-score.tsv”), i.e. sequence name, score for each of the 5 classifiers, maximum score for this sequence, group yielding this maximum score, sequence length, number of hallmark gene(s) for the maximum score group, percentage of viral and cellular genes, along with an additional column (“Manual inspection notes”) indicating the conclusions from the expert curators. The DRAM-v annotation tabs column headers correspond to the default DRAM-v output.

## Data Availability

Source code of VirSorter2 is available at bitbucket (https://bitbucket.org/MAVERICLab/VirSorter2). VirSorter2 can be installed via bioconda [71] (https://anaconda.org/bioconda/virsorter). A web version can be accessed at CyVerse/iVirus (https://de.cyverse.org/de). The databases used in VirSorter2, high quality non-RefSeq genomes used for training classifier and benchmark (except for ssDNA viral genomes [Varsani, unpublished data]), benchmark result tables (recall, precision, and F1 score), and ocean virome contig data used for manual curation and its VirSorter2 and VirSorter results are available at zenodo (https://zenodo.org/record/4297575; see instruction in README file). The VirSorter2 database can be automatically downloaded through VirSorter2 “setup” subcommand.
