# Supplementary material for: VirSorter2: a multi-classifier, expert-guided approach to detect diverse DNA and RNA viruses
Source: Microbiome. 2021 Feb 1;9:37. doi: 10.1186/s40168-020-00990-y (PMC7852108; doi:10.1186/s40168-020-00990-y)
Supplement: Supplementary file 2 — Additional file 1: Figure S1. Recall comparisons of tools on dsDNA phages from different data sources. VirSorter2 consistently has comparable or better performance than existing tools in identifying dsDNA phages. Genome fragments of different lengths (x-axis) are generated from genomes in the order Caudovirales in NCBI Viral RefSeq (A), proviruses extracted from microbial genomes in NCBI RefSeq (B) and other sources (C) (described in the “Training classifier” part of the Method section). An equal number (50) of viral and non-viral (archaea and bacteria, fungi and protozoa, and plasmids) genome fragments were combined as an input for the tested tools. Error bars show 95% confidence intervals over five replicates (100 sequences each as described above). Recall is used as the metric (y-axis) to compare tools. The dotted line is y = 0.8. Figure S2. Precision comparisons of tools on dsDNA phages from different data sources. Genome fragments of different lengths (x-axis) are generated from genomes in the order Caudovirales in NCBI Viral RefSeq (A), proviruses extracted from microbial genomes in NCBI RefSeq (B) and other sources (C) (described in the “Training classifier” part of the Method section). An equal number (50) of viral and non-viral (archaea and bacteria, fungi and protozoa, and plasmids) genome fragments were combined as an input for the tested tools. Error bars show 95% confidence intervals over five replicates (100 sequences each as described above). Precision is used as the metric (y-axis) to compare tools. The dotted line is y = 0.8. Figure S3. Tool performances with viral sequences having < 25% of the genes annotated as viral. Genome fragments of different lengths (x-axis) were generated from Caudovirales genomes from both NCBI RefSeq genomes and other sources. Only data sources with > 10 viral sequences that have < 25% genes annotated as viral were kept. Then equal numbers (50) of viral and non-viral (archaea and bacteria, fungi and protozoa, and plasmids [file 40168_2020_990_MOESM2_ESM.docx]

**Supplemental materials**


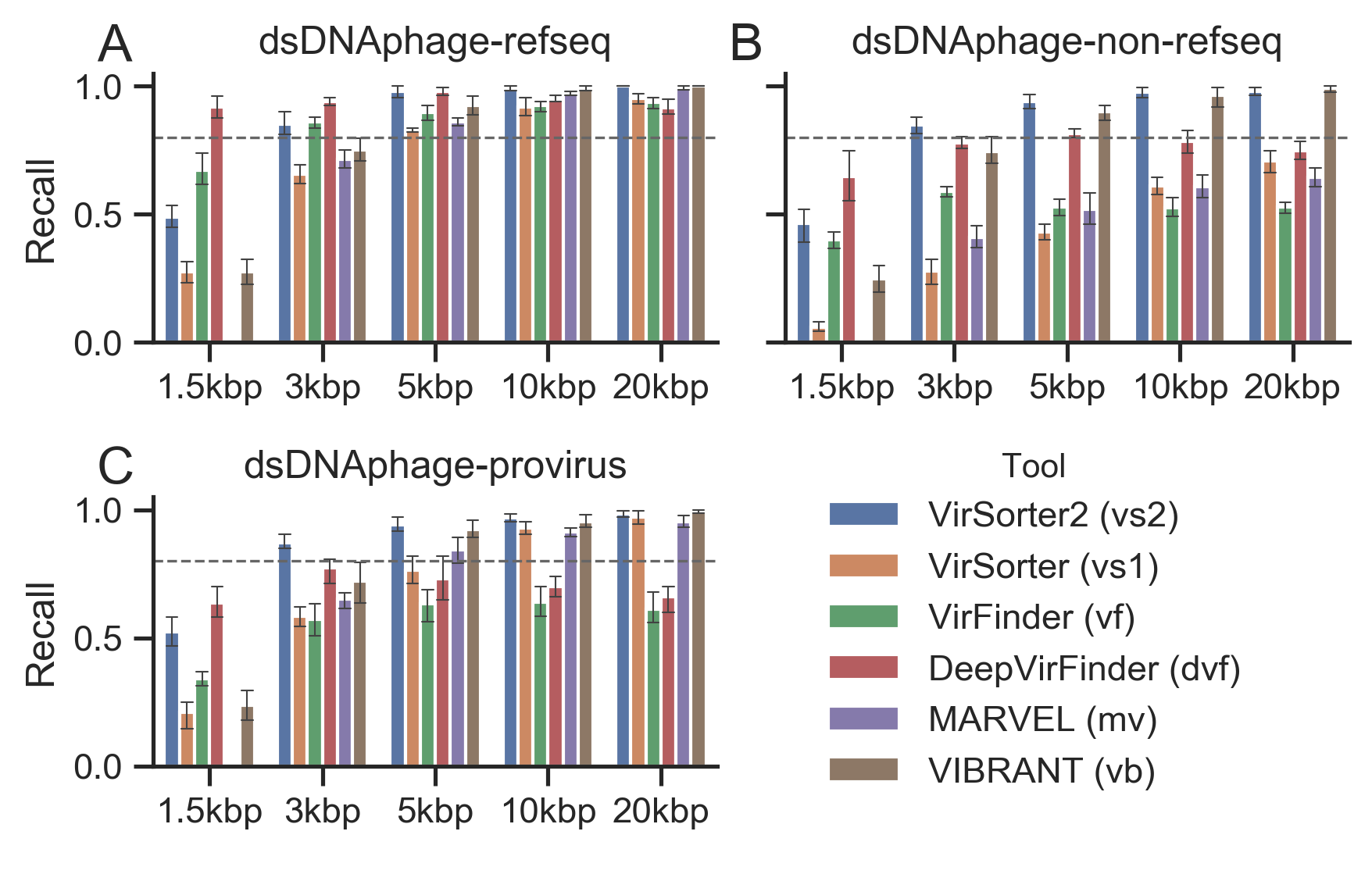


**Figure S1.** Recall comparisons of tools on dsDNA phages from different data sources. VirSorter2 consistently has comparable or better performance than existing tools in identifying dsDNA phages. Genome fragments of different lengths (x-axis) are generated from genomes in the order *Caudovirales* in NCBI Viral RefSeq **(A)**, proviruses extracted from microbial genomes in NCBI RefSeq **(B)** and other sources **(C)** (described in the “training classifier” part of the Method section). An equal number (50) of viral and non-viral (archaea and bacteria, fungi and protozoa, and plasmids) genome fragments were combined as an input for the tested tools. Error bars show 95% confidence intervals over five replicates (100 sequences each as described above). Recall is used as the metric (y-axis) to compare tools. The dotted line is y = 0.8.


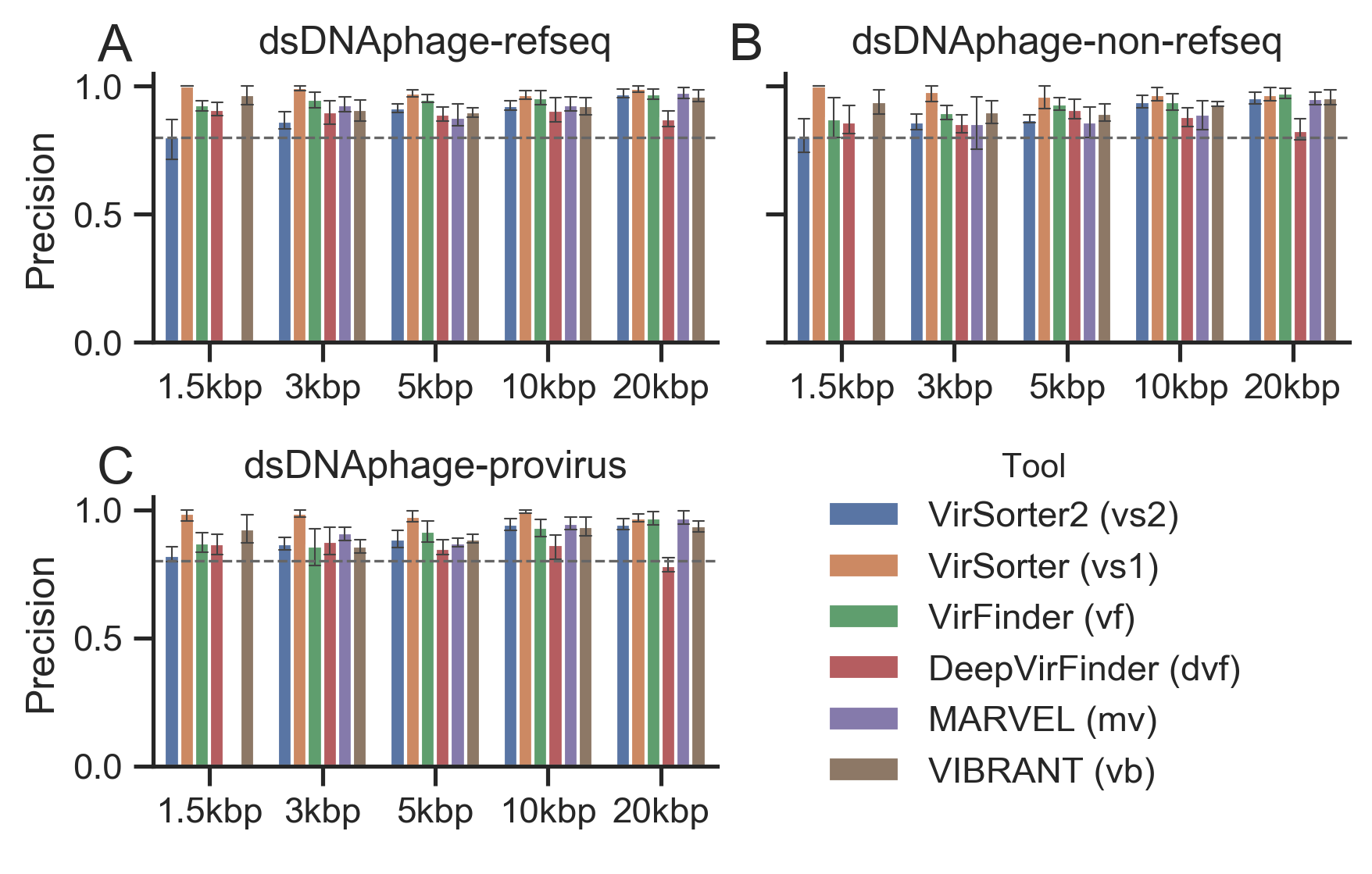


**Figure S2.** Precision comparisons of tools on dsDNA phages from different data sources. Genome fragments of different lengths (x-axis) are generated from genomes in the order *Caudovirales* in NCBI Viral RefSeq **(A)**, proviruses extracted from microbial genomes in NCBI RefSeq **(B)** and other sources **(C)** (described in the “training classifier” part of the Method section). An equal number (50) of viral and non-viral (archaea and bacteria, fungi and protozoa, and plasmids) genome fragments were combined as an input for the tested tools. Error bars show 95% confidence intervals over five replicates (100 sequences each as described above). Precision is used as the metric (y-axis) to compare tools. The dotted line is y = 0.8.


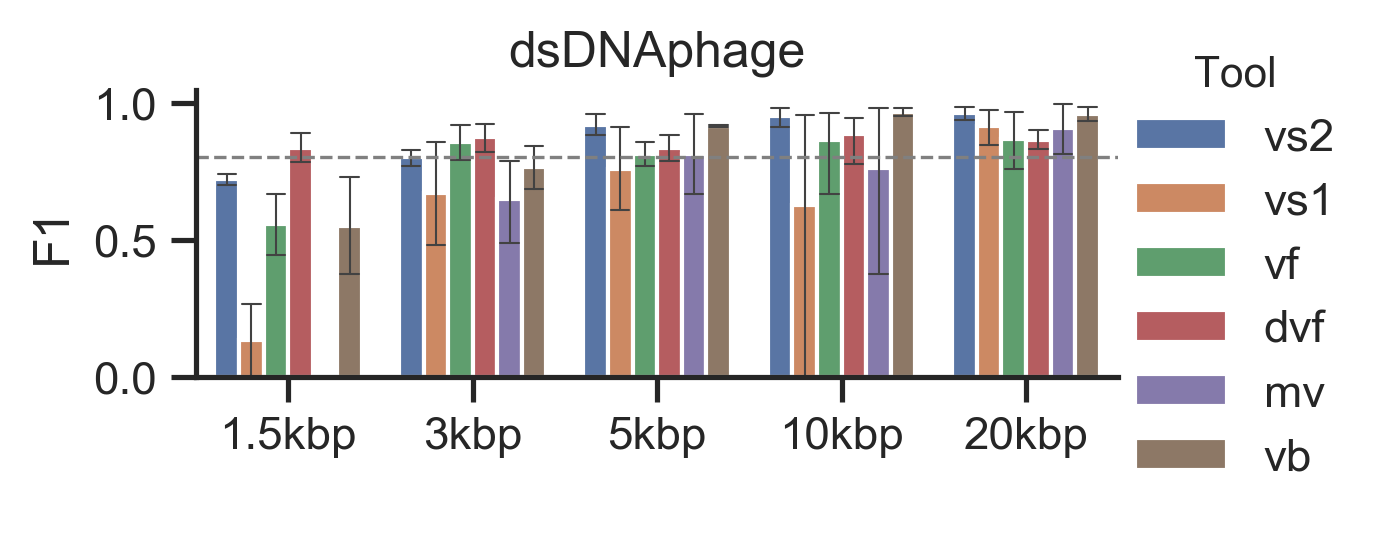


**Figure S3.** Tool performances with viral sequences having < 25% of the genes annotated as viral. Genome fragments of different lengths (x-axis) were generated from *Caudovirales* genomes from both NCBI RefSeq genomes and other sources. Only data sources with > 10 viral sequences that have < 25% genes annotated as viral were kept. Then equal numbers (50) of viral and non-viral (archaea and bacteria, fungi and protozoa, and plasmids) genome fragments were combined as an input for the tested tools. F1 score is used as the metric (y-axis) to compare tools. vs2 = VirSorter2; vs1 = VirSorter; vf = VirFinder; dvf = DeepVirFinder; mv = MARVEL; vb = VIBRANT.


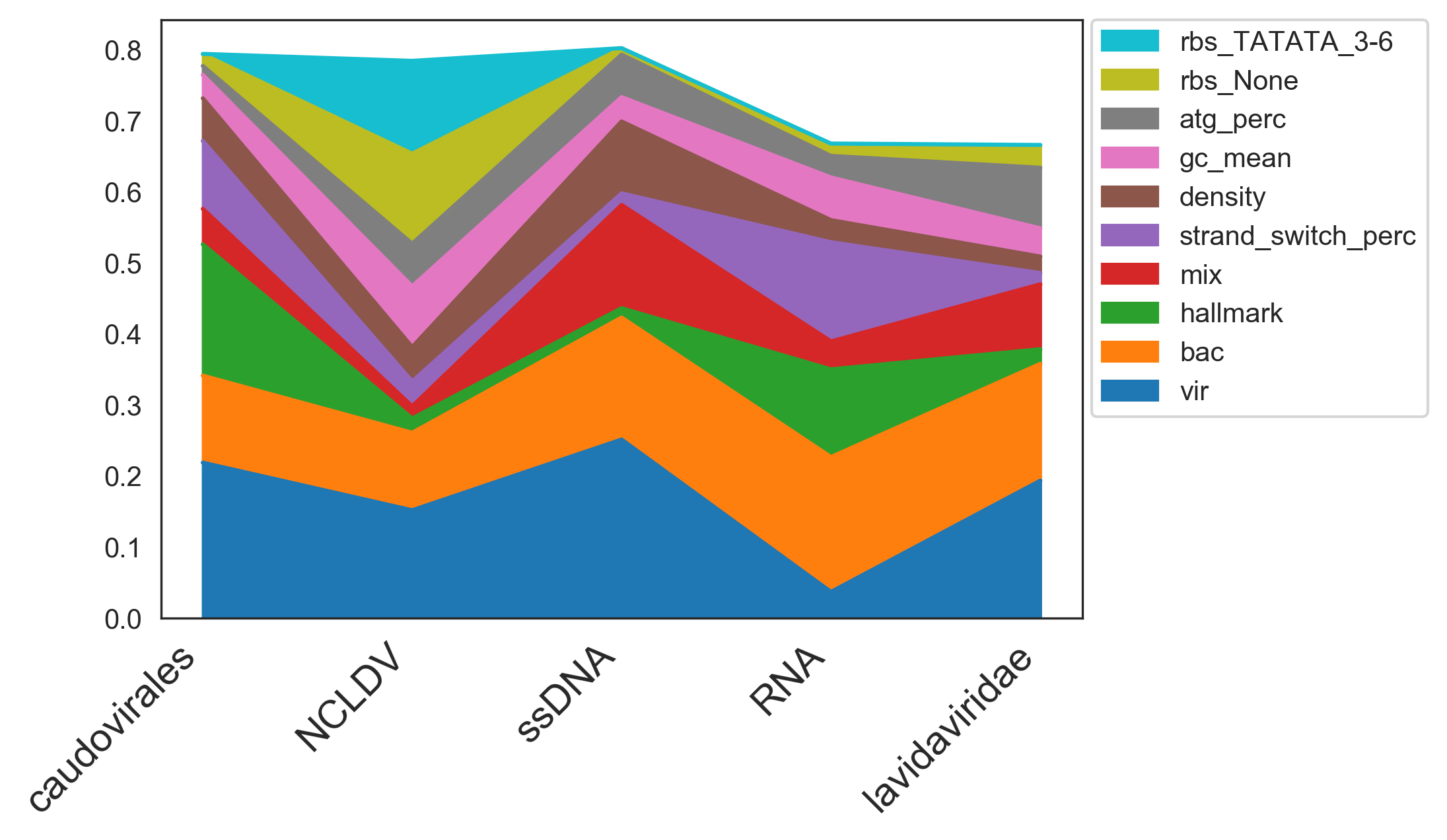


**Figure S4.** Importance of different features for viral sequence identification across viral groups. The y-axis shows the relative contribution of individual features in separating the training viral and nonviral (bacterial and archaea, fungi and protozoa, and plasmid) data (total is 1), provided by the Random Forest classifier after processing training data, and based on the F1 score. Top four features from each viral group (10 in total) are shown. In the features (color), “vir” (% of viral genes) is calculated as the percent of genes annotated as viral (best hit to viral HMMs) of all genes; “bac” (% of bacterial genes) is calculated as the percent of genes annotated as bacterial (best hit to bacterial HMMs) of all genes; “hallmark” (hallmark gene count) is the count of hallmark genes in a viral sequence; “mix” (% of mixed genes) is calculated as the percent of genes with best hit to HMMs not specific to virus or non-virus; “Strand_switch_perc” (Strand switching frequency) is the percent of genes located on a different strand from the previous gene (scanning from 5′ to 3′ in the + strand); “density” (Gene density) is the average number of genes in every 1000 bp sequence (total number of genes divided by contig length and then multiply by 1000); “gc_mean” (Mean GC content) is the mean of GC content of all genes in a contig; “atg_perc” (% of ATG as start codon) is the percent of genes with ATG as a starting codon; “rbs_None” is the percent of ribosomal binding sites (RBS) with no motif detected; “rbs_TATATA_3-6” is the percent of RBS with “TATATA_3-6” motif.


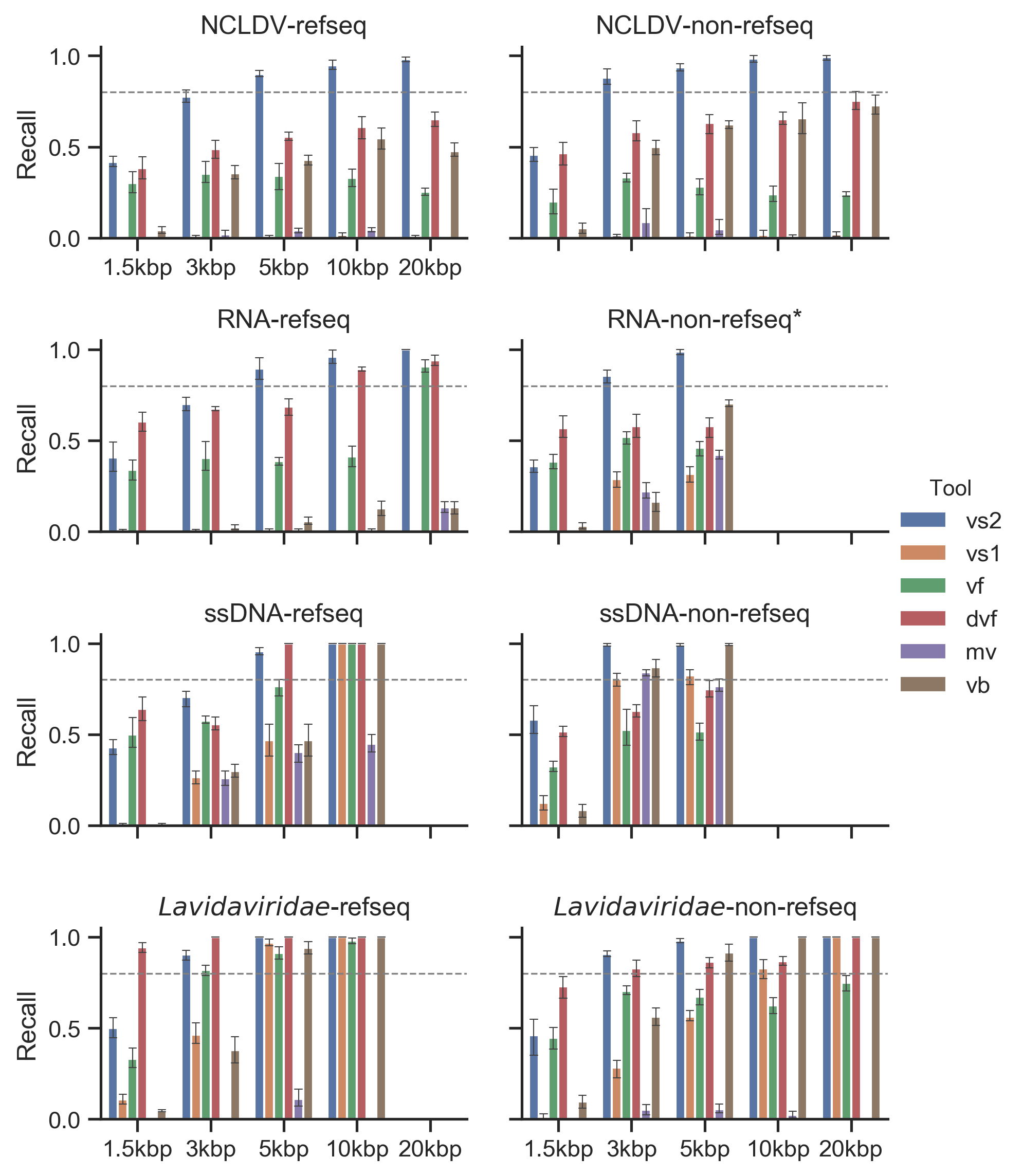


**Figure S5.** Recall comparisons of tools on different viral groups (other than dsDNA phage) from different data sources. Genome fragments of different lengths (x-axis) are generated from NCBI RefSeq (“refseq”) genomes in each viral group and other sources (“non-refseq”). Then equal numbers (50) of viral and non-viral (archaea and bacteria, fungi and protozoa, and plasmids) genome fragments were combined as input for tools. Recall was used as the metric (y-axis) to compare tools. The dotted horizontal line is y = 0.8. vs2 = VirSorter2; vs1 = VirSorter; vf = VirFinder; dvf = DeepVirFinder; mv = MARVEL; vb = VIBRANT.


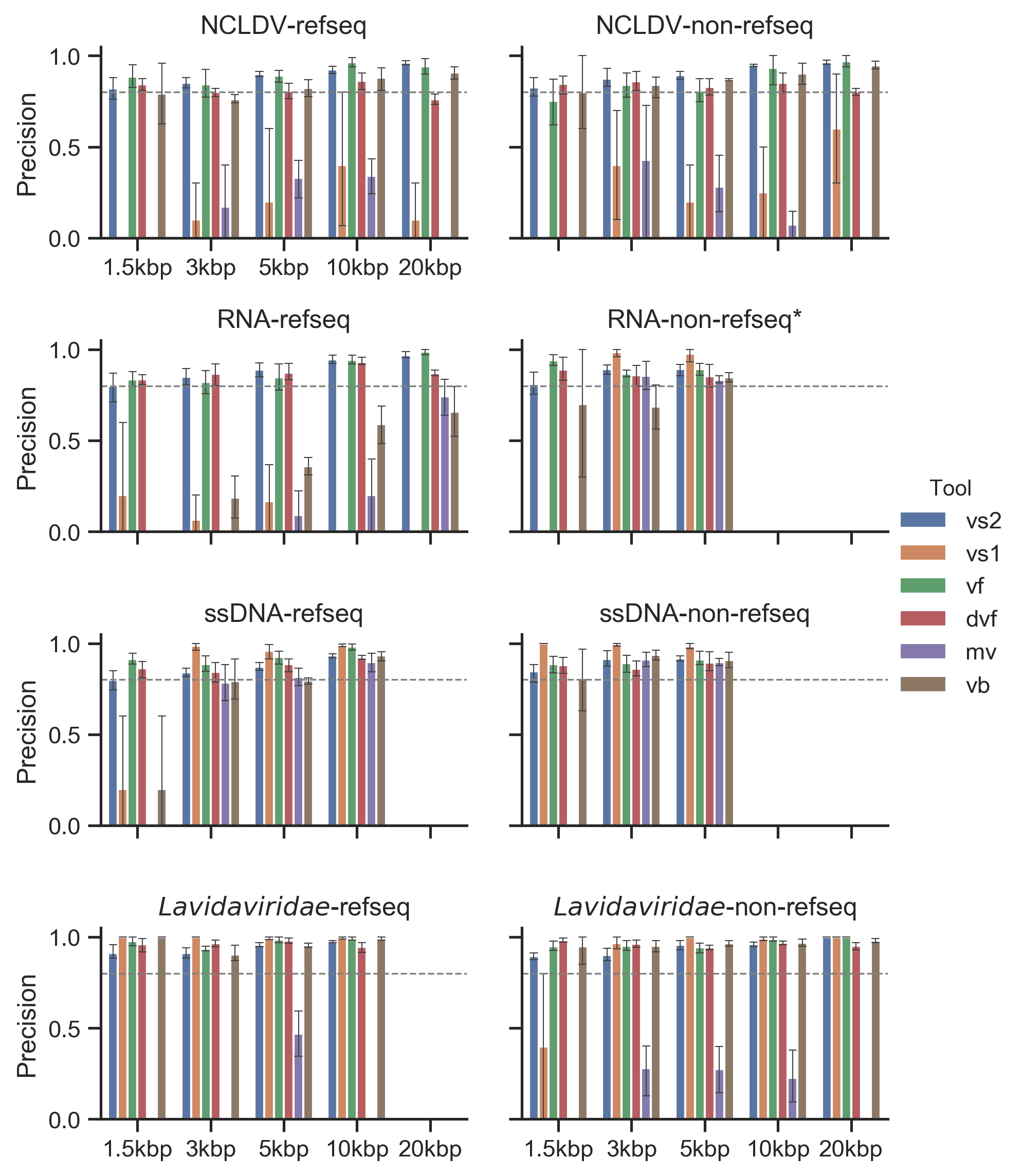


**Figure S6.** Precision comparisons of tools on different viral groups (other than dsDNA phage) from different data sources. Genome fragments of different lengths (x-axis) are generated from NCBI RefSeq (“refseq”) genomes in each viral group and other sources (“non-refseq”). Then equal numbers (50) of viral and non-viral (archaea and bacteria, fungi and protozoa, and plasmids) genome fragments were combined as input for tools. Precision was used as the metric (y-axis) to compare tools. The dotted horizontal line is y = 0.8. vs2 = VirSorter2; vs1 = VirSorter; vf = VirFinder; dvf = DeepVirFinder; mv = MARVEL; vb = VIBRANT.


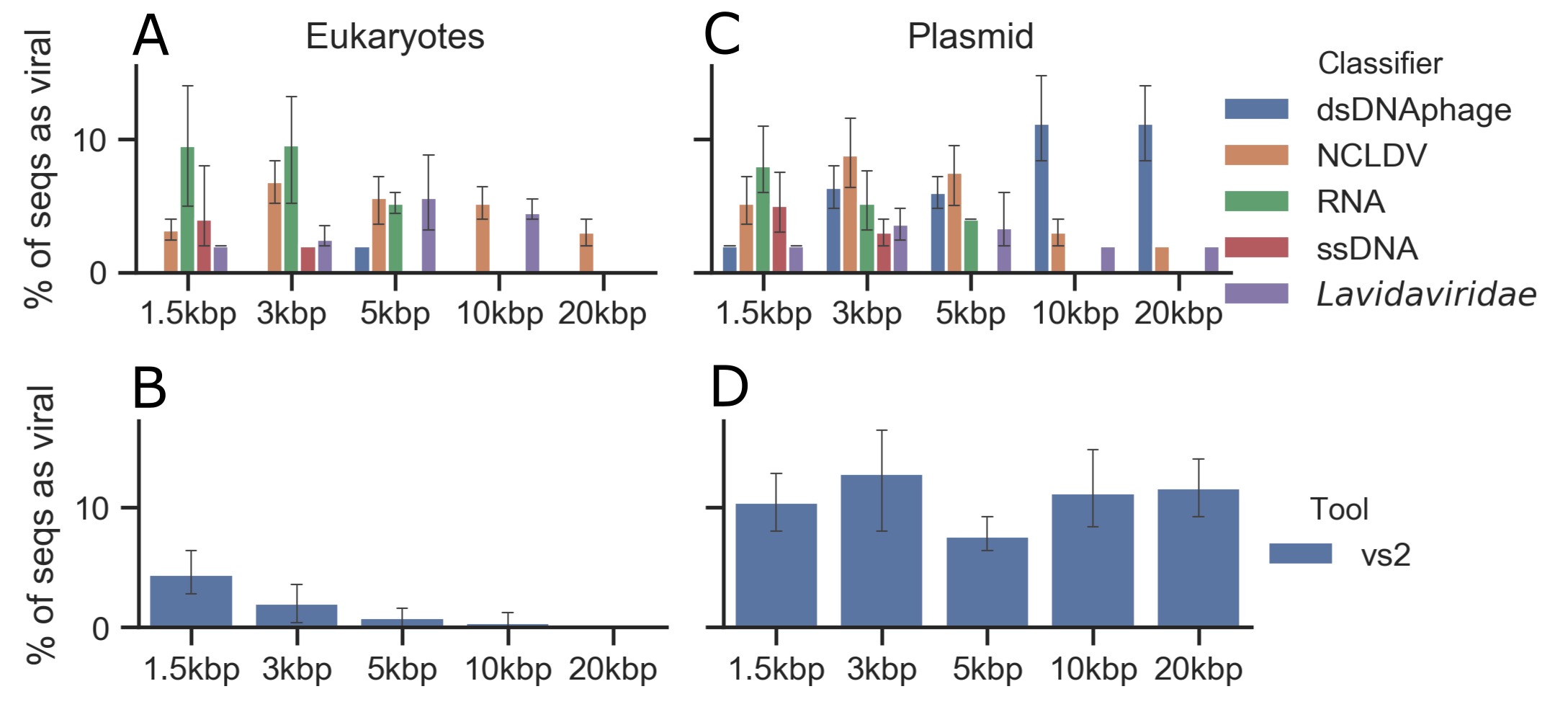


**Figure S7.** False positives by VirSorter2 on eukaryotes and plasmids. Genome fragments (50) of different lengths (x-axis) are generated from eukaryotic genomes (fungi and protozoa) in NCBI RefSeq, and plasmids. Percent of genome fragments classified as viral was used as the metric (y-axis) to evaluate tools. Plot **A** and **C** show contribution of each classifier (color) to total false positives in VirSorter2 (as shown in Fig. 4) for eukaryotes and plasmid respectively. Plot **B** and **D** show the total false positive in VirSorter2 after excluding NCLDV, RNA, and *Lavidaviridae* classifiers. vs2 = VirSorter2.


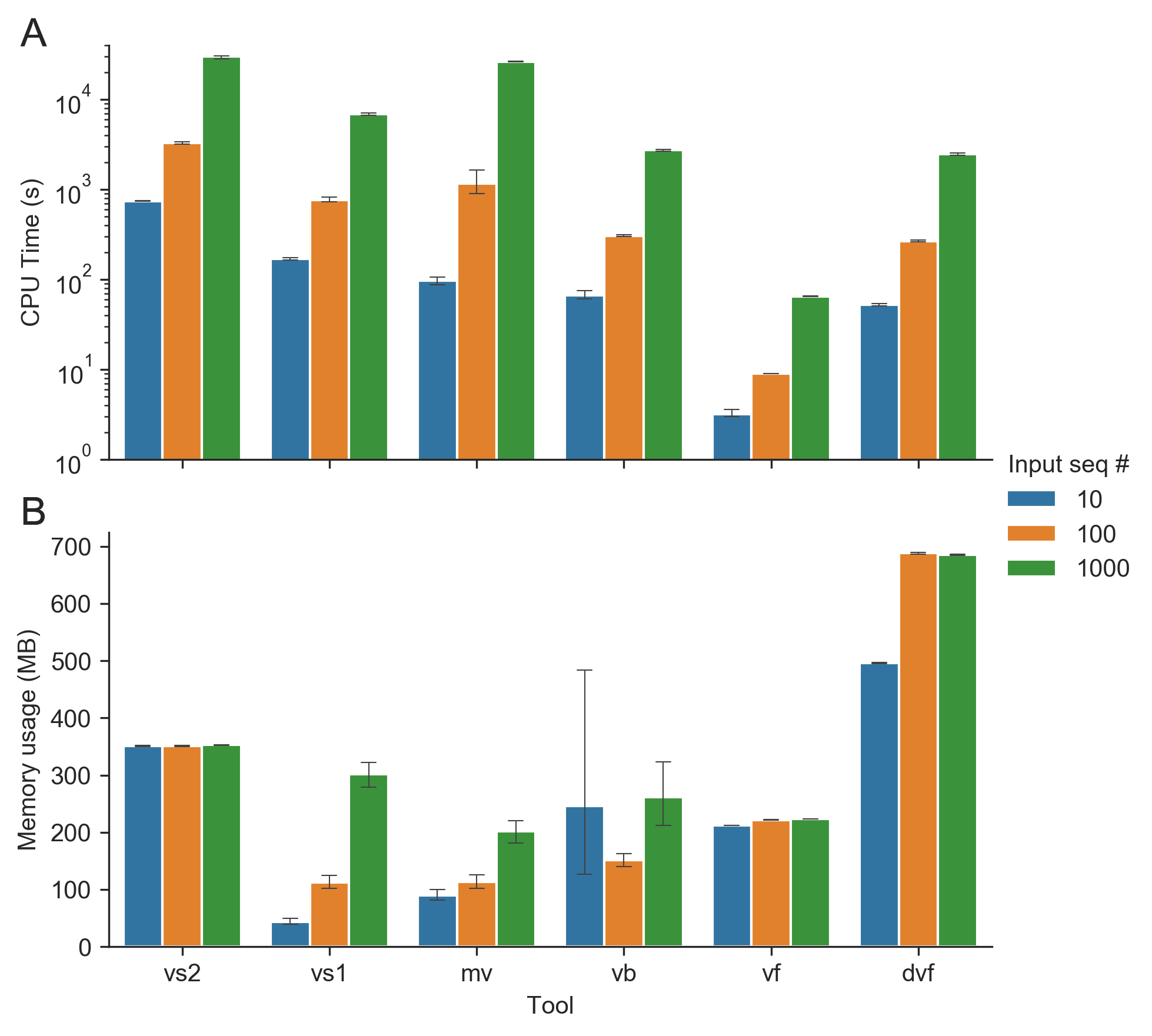


**Figure S8.** CPU time and peak memory comparison among tools across data sizes. Tools were run on different input sizes of 10, 100, 1000 sequences with 10 kb in length. Plot **(A)** shows all tools scale nearly linearly with data size, and **(B)** shows peak memory usage of all tools are <1 GB. VirSorter2 and VirFinder peak memory usage stay nearly constant. vs2 = VirSorter2; vs1 = VirSorter; vf = VirFinder; dvf = DeepVirFinder; mv = MARVEL; vb = VIBRANT.


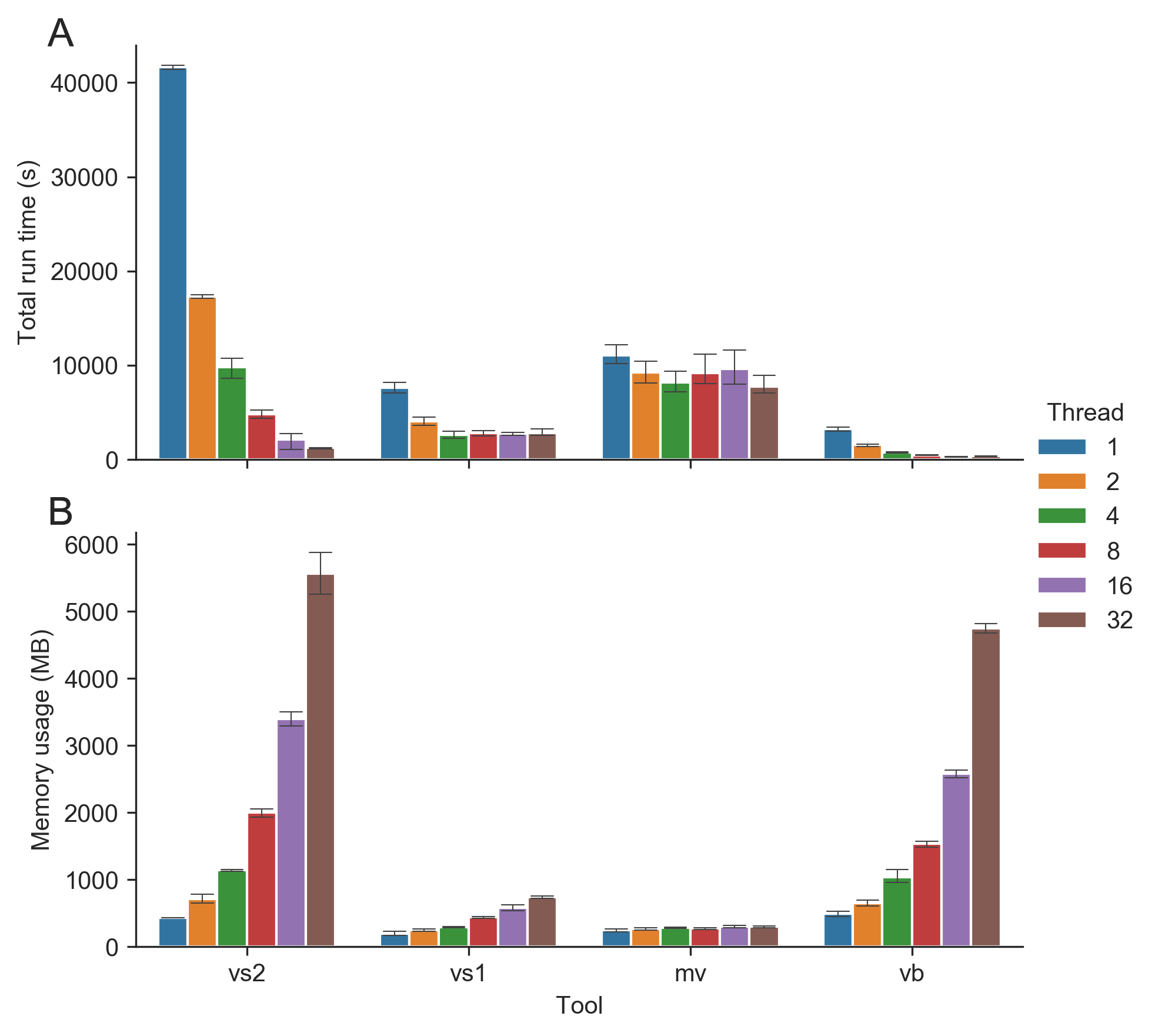


**Figure S9.** Multi-threading efficiency comparison among tools. Tools were run on 1000 sequences with 10 kb in length. Plot **(A)** shows VirSorter2 and VIBRANT have the best multi-threading efficiency, i.e. total run time decreases nearly linearly with the number of threads used. VirSorter can not use more than four threads. MARVEL’s multi-threading option does not significantly reduce run time. Plot **(B)** shows VirSorter2, VirSorter and VIBRANT memory usage increases with the number of threads used, with VirSorter2 and VIBRANT increasing at a higher rate than VirSorter. MARVEL’s memory usage stayed constant. vs2 = VirSorter2; vs1 = VirSorter; mv = MARVEL; vb = VIBRANT.


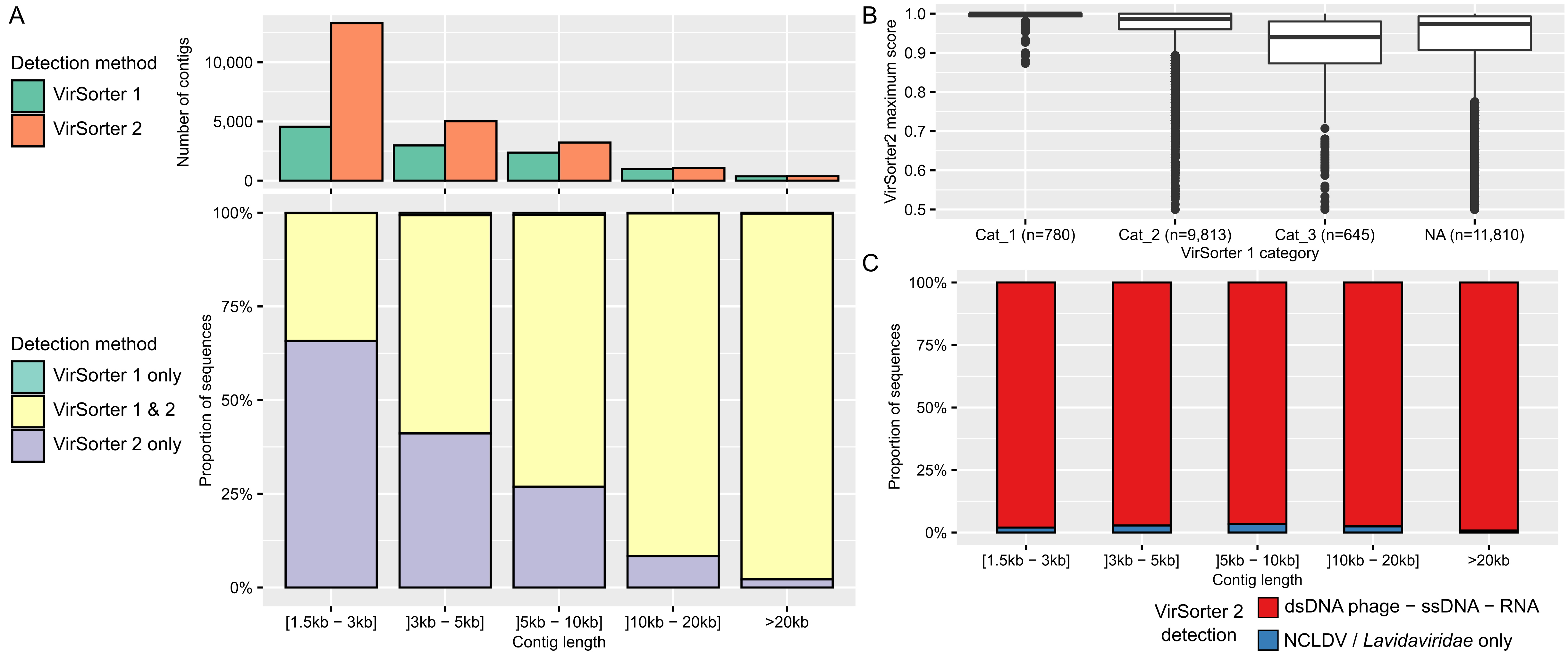


**Figure S10**: Overview of VirSorter2 results for *Tara* Oceans virome 85_SRF. **A.** Detection of viral contigs via VirSorter 1.0.4 and VirSorter 2.0.beta by contig size. The top panel displays the total number of viral contigs identified in each size class, while the bottom panel indicates the overlap between these predictions. **B.** Distribution of VirSorter 2.0.beta score (maximum score across all 5 classifiers, y-axis) for *Tara* Oceans virome 85_SRF sequences, according to the confidence category estimated by VirSorter1 (x-axis). “NA'' indicates contigs that were not detected as viral by VirSorter1. VirSorter 2.0.beta detections were based on a minimum score cutoff of 0.5. **C.** Proportion of sequences from Tara Oceans virome 85_SRF detected as viral based on the dsDNAphage, RNA, and/or ssDNA model(s) (red) or detected based on the NCLDV or *Lavidaviridae* classifiers only, by size class (x-axis).

**Table S1.** Summary statistics of the genomes used for training VirSorter2 classifiers of different viral groups from RefSeq and non-RefSeq sources. “Genome #” is the number of genomes. “Min size”, “Median size”, and “Max size” are the minimum, median, and maximum of genome sizes per each viral group.

| **Group** | **Source** | **Genome #** | **Min size(bp)** | **Median size (bp)** | **Max size (bp)** |
| --- | --- | --- | --- | --- | --- |
| NCLDV | non-refseq | 501 | 100409 | 325579 | 1406447 |
| NCLDV | refseq | 136 | 73689 | 198103.5 | 2473870 |
| RNA | non-refseq | 365 | 2731 | 3810 | 5693 |
| RNA | refseq | 3783 | 1005 | 8974 | 41178 |
| dsDNAphage | non-refseq | 25635 | 15005 | 44469 | 570671 |
| dsDNAphage | provirus | 2878 | 15098 | 42382 | 136518 |
| dsDNAphage | refseq | 2442 | 11624 | 50879.5 | 497513 |
| *lavidavirida*e | non-refseq | 194 | 17322 | 24236 | 42373 |
| *lavidaviridae* | refseq | 7 | 17276 | 19527 | 29767 |
| ssDNA | non-refseq | 10010 | 1000 | 4199 | 10865 |
| ssDNA | refseq | 1475 | 1000 | 2744 | 24893 |
