# Supplementary material for: VirSorter2: a multi-classifier, expert-guided approach to detect diverse DNA and RNA viruses
Source: Microbiome. 2021 Feb 1;9:37. doi: 10.1186/s40168-020-00990-y (PMC7852108; doi:10.1186/s40168-020-00990-y)
Supplement: Supplementary file 3 — Additional file 2. Case-study: identifying viral contig from a Tara Oceans virome dataset [file 40168_2020_990_MOESM3_ESM.docx]

**Supplementary Text: VirSorter2: A multi-classifier, expert-guided approach to detect diverse DNA and RNA viruses**

Jiarong Guo^1^, Ben Bolduc^1^, Ahmed A. Zayed^1^, Arvind Varsani^2,3^, Guillermo Dominguez-Huerta^1^, Tom O. Delmont^4^, Akbar Adjie Pratama^1^, M Consuelo Gazitúa^5^, Dean Vik^1^, Matthew B. Sullivan^1,6,7 *^, Simon Roux^8 *^

1. Department of Microbiology, Ohio State University, Columbus, OH 43210, USA

2. The Biodesign Center for Fundamental and Applied Microbiomics, Center for Evolution and Medicine, School of Life Sciences, Arizona State University, Tempe, AZ 85287, USA

3. Structural Biology Research Unit, Department of Integrative Biomedical Sciences, University of Cape Town, Observatory, Cape Town 7701, South Africa

4. Génomique Métabolique, Genoscope, Institut François Jacob, CEA, CNRS, Univ Evry, Université Paris-Saclay, 91057 Evry, France

5. Viromica, Santiago, 7870582, Chile

6. Civil, Environmental and Geodetic Engineering, Ohio State University, Columbus, OH 43210

7. Center of Microbiome Science, Ohio State University, Columbus, OH 43210, USA

8. DOE Joint Genome Institute, Lawrence Berkeley National Laboratory, Berkeley, CA 94720

* Correspondence to: Matthew B. Sullivan (sullivan.948@osu.edu) Simon Roux (sroux@lbl.gov)

**Supplementary Text**

Case-study: identifying viral contig from a *Tara* Oceans virome dataset

*VirSorter2 prediction of viral sequences in* Tara *Oceans virome 85_SRF*

In order to illustrate how to read and interpret VirSorter2 results, we will describe here viral sequence prediction results obtained for one of the *Tara* Oceans viromes (85_SRF, TARA_R100001369, [[1]](https://www.zotero.org/google-docs/?OTJFIC)), and contrast them with results obtained with VirSorter 1.0.4 on the same sequences. *Tara* Oceans virome 85_SRF dataset includes 1,199,077 contigs (46,002 of them longer than 1.5kb) assembled from short-read viral metagenome, which we expect the majority to originate from dsDNA viral genomes. We also expect relatively few “giant viruses” (NCLDV) to be sequenced as part of this virome because of the combination of filters used [[1]](https://www.zotero.org/google-docs/?7Kc6o3). Hence, we will focus here on viral sequence predictions corresponding to contigs entirely viral (which represent > 99% of the predictions for both VirSorter 1 and 2), and interpret these results from the perspective of a researcher interested in the dsDNA bacteriophage and archaeovirus community.

VirSorter 2.0.beta was used to predict viral sequences from 85_SRF contigs with default parameters, i.e. “*virsorter run -i 85_SRF_contigs.fna -w 85_SRF_VS2results/*”. With default parameters, VirSorter 2.0.beta will use the 5 available classifiers (dsDNAphage,NCLDV,RNA,ssDNA, and *lavidaviridae*), and a minimum score of 0.5. The main results are presented in a score table: “final-viral-score.tsv” (Additional file 1). For each sequence, this table includes the score across each model, along with columns indicating the highest score for the sequence, the model yielding this highest score, the number of hallmark gene(s) for this highest score model, and the percentage of genes annotated as viral or cellular in this sequence. Note that these two percentages do not necessarily sum to 100%, the remainder being predicted genes that can not be confidently annotated as viral or cellular. Since this table also only includes sequences predicted as viral, all sequences display a minimum score of 0.5 in the “max_score” column, consistent with the default 0.5 cutoff (Additional file 1). Short sequences with less than 2 complete genes and predicted as viral based on the presence of hallmark gene(s) are identified with an “lt2gene” tag, and have an “nan” score for all models.

*Comparison of VirSorter2 and VirSorter results on virome 85_SRF*

Overall, and considering only sequences predicted as entirely viral, VirSorter 2.0.beta identifies 22,973 viral contigs using these default parameters. This represents nearly a two-fold increase compared to the 14,183 viral contigs identified with VirSorter 1.0.4 (categories 1, 2, and 3). This increase is mostly associated with short contigs: while > 97% of the viral contigs ≥20kb are identified by both tools, 66% of viral contigs < 3kb are only identified by VirSorter2 (Fig. S10A). This illustrates a known limitation of VirSorter with short (< 10kb) contigs [[2]](https://www.zotero.org/google-docs/?XiQanc), and how VirSorter2 is better able to recover these short contigs, as expected from our benchmarks (Fig. 3B). Importantly, only 0.3% of sequences (75 of 23,048 total) are exclusively predicted as viral with VirSorter. This shows how VirSorter2 essentially recovers the same viral contigs as VirSorter, and then expands to viral contigs which so far represented “blind spots” for VirSorter.

While VirSorter associated each prediction to a “confidence category” from 1 to 3, 1 being the most confident predictions, and 3 the less confident, this has now been replaced in VirSorter2 by the scores obtained with the different random forest classifiers. To guide users in translating the former categories into scores, we analyzed the distribution of VirSorter2 “max_score” (i.e. the score of the highest scoring model) for each of VirSorter confidence category and for sequences not predicted as viral by VirSorter 1.0.4 (Fig. S10B). As expected, VirSorter2 score decreases from high-confidence category 1 sequences (median score = 1) to low-confidence category 3 sequences (median score = 0.94). Hence, for users analyzing datasets that may contain a high number of “challenging” sequences (e.g. plasmids and other mobile genetic elements) and only few viruses, for which VirSorter recommended analyzing only sequences of category 1 and 2, a minimum score of 0.95 in VirSorter2 would likely correspond to the equivalent stringency. We note however that in the case of *Tara* Oceans 85_SRF virome, such a strict threshold would discard ~ 28% of the viral predictions. Based on our current experience and manual inspection (see below), we believe the default cutoff of 0.5 is applicable to most metagenomes and does not require to be finely tuned or refined with each new dataset.

*Manual inspection of novel VirSorter2 detections from virome 85_SRF*

To further verify that the new predictions in VirSorter 2.0.beta compared to VirSorter 1.0.4 are likely viral contigs, we randomly selected 120 contigs newly predicted, including 60 ≥ 5kb and 60 < 5kb, performed an in-depth functional annotation of these contigs using DRAM-v [[3]](https://www.zotero.org/google-docs/?oYFkaT), and manually inspected these contigs for signs of cellular contamination (Additional file 2). This manual inspection was performed by different co-authors with expertise in viral metagenomics, with the contigs divided in batches so that 15 contigs in each size class would be inspected by 2 different curators. Signs of potential contamination included (i) the contig only includes gene typically associated with plasmids or other mobile genetic element, (ii) the contig includes genes associated with non-viral functions or metabolisms, i.e. features that have so far not been described in phage or viral genomes, or (iii) atypical cds prediction such as low gene density or prediction of short (< 30 amino acid) cds only. All these features are known to be challenging to identify by automated viral sequence prediction tools, and lead to false-positive calls [[2]](https://www.zotero.org/google-docs/?KsIbH6). Experts assigned each sequence as “likely viral”, “unsure”, or “likely not viral”, based on these features and their experience.

For short contigs (< 5kb), only 2 of the 60 randomly selected contigs were identified as likely contaminant by the curators. This confirms that these short contigs which were not detected by VirSorter are overwhelmingly genuine viral contigs, for which VirSorter recall is low [[2]](https://www.zotero.org/google-docs/?vxEWjJ). A ratio of ~ 3% of false-positive calls is also consistent with our benchmarks based on simulated metagenomes (Fig. S2). For long contigs (≥ 5kb), the curators identified 7 contigs as likely contaminants, i.e. an estimated false-positive rate of ~ 12% (Additional file 2). Two approaches are available to avoid these erroneous detections. First a user could use a higher score threshold: here, using a minimum score of 0.8 would remove 4 of the 7 likely contaminants (false-positive rate of ~ 5%), however this higher score threshold would also remove 7 likely genuine sequences (i.e. 13%). Another approach would be to ignore some of VirSorter2 classifiers, namely the NCLDV and *lavidaviridae.* Benchmarks from simulated data suggested that these two models were responsible for a substantial fraction of the false-positive detections (Supplementary Fig. S8). Here, 6 of the 7 sequences identified as likely contaminants only displayed a significant score (i.e. ≥ 0.5) with one of these 2 classifiers (NCLDV or *lavidaviridae*), and would thus not have been called viral if the user had only used a combination of dsDNAphage, RNA, and ssDNA classifiers. Further, this approach would only remove 1 likely genuine sequence while yielding a false-positive rate of 2%. These numbers are consistent with expectations based on the type of sample processed that most of the sequences would likely belong to dsDNA bacteriophages or archaeoviruses, and across all size classes, as sequences detected with the NCLDV or *lavidaviridae* classifiers represent < 4% of the predicted viral contigs. Below, we describe how a user could obtain a refined VirSorter2 final output, including a fasta file of predicted viral sequences and the score table, using one of these two approaches (increasing minimum score or restricting the number of classifiers considered), using an existing VirSorter2 output folder.

*Adjusting VirSorter2 output to maximize precision and recall for specific viral groups*

By default, VirSorter 2.0.beta uses a minimum score cutoff of 0.5 and uses the 5 available classifiers (dsDNAphage,NCLDV,RNA,ssDNA, and *lavidaviridae*). To increase the minimum score cutoff to 0.8 for *Tara* Oceans 85_SRF virome (see above), a user would simply need to run the following “classify” command: “*virsorter run -w 85_SRF_VS2results/ --min-score 0.8 classify*”. This will use all the annotation and results from the random forest classifiers previously generated, and only reassign the sequences as viral or non-viral using the new criteria. Similarly, in order to consider only a subset of the classifiers (dsDNAphage, RNA, and ssDNA) and only include contigs predicted as entirely viral, a user would run the following “classify” command: “*virsorter run -w 85_SRF_VS2results/ --include-groups dsDNAphage,RNA,ssDNA --provirus-off classify*”. Note that the “classify” task can be used to change minimum score, hallmark gene requirement, viral gene requirement, and list of models used, in any direction, i.e. either restricting or broadening these. Hence, a user could also check with the restricted run (“*--include-groups dsDNAphage,RNA,ssDNA --min-score 0.8*”), and then reclassify with (“*--include-groups NCLDV,lavidaviridae,dsDNAphage,RNA,ssDNA --min-score 0.5*”) to expand the predicted viral sequences to all models and all cases with score ≥ 0.5. While the “classify” command overwrites previous results by default, the “--label” option can be used to avoid that and keep both sets of results in the same VirSorter2 directory.

Importantly, the exact options and output files described here may see minor modifications in future versions of VirSorter2, and users should refer to the documentation corresponding to the VirSorter2 version they are working with. Nevertheless, the interpretation of VirSorter2 results will remain essentially the same, and the guiding principles will still be applicable to these future versions.

*References:*

1. Gregory AC, Zayed AA, Conceição-Neto N, Temperton B, Bolduc B, Alberti A, et al. Marine DNA Viral Macro- and Microdiversity from Pole to Pole. Cell. 2019;177:1109-1123.e14.

2. Roux S, Enault F, Hurwitz BL, Sullivan MB. VirSorter: mining viral signal from microbial genomic data. PeerJ. 2015;3:e985.

3. Shaffer M, Borton MA, McGivern BB, Zayed AA, Rosa SLL, Solden LM, et al. DRAM for distilling microbial metabolism to automate the curation of microbiome function. bioRxiv. Cold Spring Harbor Laboratory; 2020;2020.06.29.177501.

Figure S10: Overview of VirSorter2 results for *Tara* Oceans virome 85_SRF. **A.** Detection of viral contigs via VirSorter 1.0.4 and VirSorter 2.0.beta by contig size. The top panel displays the total number of viral contigs identified in each size class, while the bottom panel indicates the overlap between these predictions. **B.** Distribution of VirSorter 2.0.beta score (maximum score across all 5 classifiers, y-axis) for *Tara* Oceans virome 85_SRF sequences, according to the confidence category estimated by VirSorter (x-axis). “NA'' indicates contigs that were not detected as viral by VirSorter. VirSorter 2.0.beta detections were based on a minimum score cutoff of 0.5. **C.** Proportion of sequences from Tara Oceans virome 85_SRF detected as viral based on the dsDNAphage, RNA, and/or ssDNA model(s) (red) or detected based on the NCLDV or *Lavidaviridae* classifiers only, by size class (x-axis).

Additional file 1: First 20 rows of the “final-viral-score.tsv” VirSorter2 output file for Tara Oceans virome 85_SRF. VirSorter 2.0.beta was used with default parameters, including all classifiers and a minimum score cutoff of 0.5. The columns include first the sequence name, the score for each of the 5 classifiers, the maximum score for this sequence, the group yielding this maximum score, the sequence length, the number of hallmark gene(s) for the maximum score group, and the percentage of viral and cellular genes.

Additional file 2: Manual inspection of contigs newly identified by VirSorter2 in Tara Oceans virome 85_SRF. The spreadsheet includes four tabs, including the list and characteristics of contigs ≥ 5kb selected for manual inspection (“Contigs ≥ 5kb - Manual inspection”), the DRAM-v annotation of these contigs (“Contigs ≥ 5kb - DRAM-v annotation”), the list and characteristics of contigs ≥ 5kb selected for manual inspection (“Contigs < 5kb - Manual inspection”), and their DRAM-v annotation (“Contigs < 5kb - DRAM-v annotation”). For the manual inspection tabs, column headers correspond to the standard output of VirSorter2 (“final-viral-score.tsv”), i.e. sequence name, score for each of the 5 classifiers, maximum score for this sequence, group yielding this maximum score, sequence length, number of hallmark gene(s) for the maximum score group, percentage of viral and cellular genes, along with an additional column (“Manual inspection notes”) indicating the conclusions from the expert curators. The DRAM-v annotation tabs column headers correspond to the default DRAM-v output.
